# Supplementary material for: Pan-cancer molecular analysis of the RB tumor suppressor pathway
Source: Commun Biol. 2020 Apr 2;3:158. doi: 10.1038/s42003-020-0873-9 (PMC7118159; doi:10.1038/s42003-020-0873-9)
Supplement: Supplementary file 1 — Supplementary Information [file 42003_2020_873_MOESM1_ESM.pdf]

Study Abbreviation	Study Name	No. of Samples
LAML	Acute Myeloid Leukemia	173
ACC	Adrenocortical carcinoma	78
BLCA	Bladder Urothelial Carcinoma	407
LGG	Brain Lower Grade Glioma	514
BRCA	Breast invasive carcinoma	1082
CESC	Cervical squamous cell carcinoma and endocervical adenocarcinoma	294
CHOL	Cholangiocarcinoma	36
COAD	Colon adenocarcinoma	592
ESCA	Esophageal carcinoma	181
GBM	Glioblastoma multiforme	160
HNSC	Head and Neck squamous cell carcinoma	515
KICH	Kidney Chromophobe	65
KIRC	Kidney renal clear cell carcinoma	510
KIRP	Kidney renal papillary cell carcinoma	283
LIHC	Liver hepatocellular carcinoma	366
LUAD	Lung adenocarcinoma	510
LUSC	Lung squamous cell carcinoma	484
DLBC	Lymphoid Neoplasm Diffuse Large B-cell Lymphoma	48
MESO	Mesothelioma	87
OV	Ovarian serous cystadenocarcinoma	300
PAAD	Pancreatic adenocarcinoma	177
PCPG	Pheochromocytoma and Paraganglioma	178
PRAD	Prostate adenocarcinoma	493
SARC	Sarcoma	253
SKCM	Skin Cutaneous Melanoma	443
STAD	Stomach adenocarcinoma	412
TGCT	Testicular Germ Cell Tumors	149
THYM	Thymoma	119
THCA	Thyroid carcinoma	498
UCS	Uterine Carcinosarcoma	57
UCEC	Uterine Corpus Endometrial Carcinoma	527
UVM	Uveal Melanoma	80

Supplementary Figure 1: The pan-Cancer TCGA datasets and the corresponding acronym for each disease site are listed as per the TCGA. The number of samples per data set is provided in the table.

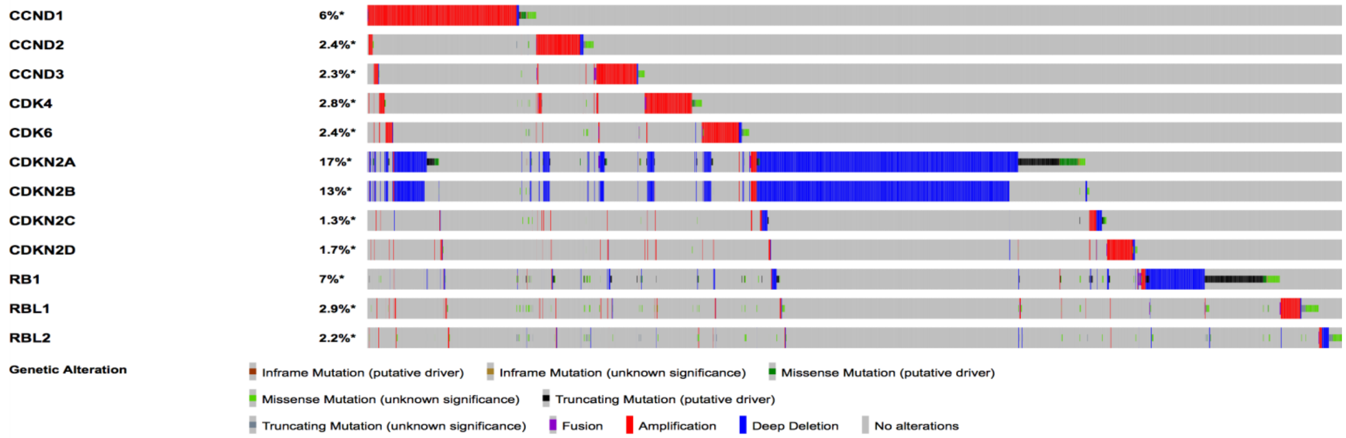

Supplementary Figure 2: An oncoprint of the indicated RB-pathway associated genes is shown in the upper panel from all cases within the pan-cancer data sets combined.

SARC

A	B	Neither	A Not B	B Not A	Both	Log2 Odds Ratio	p-Value	q-Value	Tendency
RB1	CDKN2A	152	62	38	1	<-3	<0.001	**<0.001**	**Mutual exclusivity**
RB1	CCND1	184	61	6	2	0.008	0.634	0.76	Co-occurrence
RB1	CDK4	145	63	45	0	<-3	<0.001	**<0.001**	**Mutual exclusivity**
RB1	CDK6	187	62	3	1	0.008	0.684	0.76	Co-occurrence
CDKN2A	CCND1	208	37	6	2	0.906	0.356	0.7	Co-occurrence
CDKN2A	CDK4	172	36	42	3	-1.551	0.051	0.171	Mutual exclusivity
CDKN2A	CDK6	211	38	3	1	0.888	0.49	0.7	Co-occurrence
CCND1	CDK4	203	5	42	3	1.536	0.153	0.384	Co-occurrence
CCND1	CDK6	241	8	4	0	<-3	0.879	0.879	Mutual exclusivity
CDK4	CDK6	204	45	4	0	<-3	0.454	0.7	Mutual exclusivity

BLCA

A	B	Neither	A Not B	B Not A	Both	Log2 Odds Ratio	p-Value	q-Value	Tendency
RB1	CDKN2A	152	95	150	9	<-3	<0.001	**<0.001**	**Mutual exclusivity**
RB1	CCND1	256	101	46	3	-2.597	<0.001	**<0.001**	**Mutual exclusivity**
RB1	CDK4	296	101	6	3	0.551	0.417	0.695	Co-occurrence
RB1	CDK6	294	101	8	3	0.126	0.566	0.761	Co-occurrence
CDKN2A	CCND1	229	128	18	31	1.623	<0.001	**<0.001**	**Co-occurrence**
CDKN2A	CDK4	239	158	8	1	-2.403	0.075	0.188	Mutual exclusivity
CDKN2A	CDK6	239	156	8	3	-0.8	0.314	0.627	Mutual exclusivity
CCND1	CDK4	349	48	8	1	-0.138	0.703	0.779	Mutual exclusivity
CCND1	CDK6	347	48	10	1	-0.468	0.609	0.761	Mutual exclusivity
CDK4	CDK6	386	9	11	0	<-3	0.779	0.779	Mutual exclusivity

LUAD

A	B	Neither	A Not B	B Not A	Both	Log2 Odds Ratio	p-Value	q-Value	Tendency
RB1	CDKN2A	365	37	105	0	<-3	<0.001	**0.001**	**Mutual exclusivity**
RB1	CCND1	453	35	17	2	0.607	0.411	0.654	Co-occurrence
RB1	CDK4	441	35	29	2	-0.203	0.602	0.654	Mutual exclusivity
RB1	CDK6	460	36	10	1	0.354	0.569	0.654	Co-occurrence
CDKN2A	CCND1	393	95	9	10	2.201	0.002	**0.008**	**Co-occurrence**
CDKN2A	CDK4	376	100	26	5	-0.468	0.35	0.654	Mutual exclusivity
CDKN2A	CDK6	393	103	9	2	-0.238	0.594	0.654	Mutual exclusivity
CCND1	CDK4	461	15	27	4	2.187	0.023	0.077	Co-occurrence
CCND1	CDK6	477	19	11	0	<-3	0.654	0.654	Mutual exclusivity
CDK4	CDK6	466	30	10	1	0.635	0.504	0.654	Co-occurrence

LUSC

A	B	Neither	A Not B	B Not A	Both	Log2 Odds Ratio	p-Value	q-Value	Tendency
RB1	CDKN2A	225	46	196	2	<-3	<0.001	**<0.001**	**Mutual exclusivity**
RB1	CCND1	361	46	60	2	-1.935	0.031	0.105	Mutual exclusivity
RB1	CDK4	418	48	3	0	<-3	0.723	0.803	Mutual exclusivity
RB1	CDK6	395	47	26	1	-1.629	0.212	0.425	Mutual exclusivity
CDKN2A	CCND1	247	160	24	38	1.289	<0.001	**0.005**	**Co-occurrence**
CDKN2A	CDK4	269	197	2	1	-0.551	0.616	0.803	Mutual exclusivity
CDKN2A	CDK6	254	188	17	10	-0.331	0.363	0.604	Mutual exclusivity
CCND1	CDK4	404	62	3	0	<-3	0.653	0.803	Mutual exclusivity
CCND1	CDK6	386	56	21	6	0.978	0.131	0.327	Co-occurrence
CDK4	CDK6	439	3	27	0	<-3	0.837	0.837	Mutual exclusivity

BRCA

A	B	Neither	A Not B	B Not A	Both	Log2 Odds Ratio	p-Value	q-Value	Tendency
RB1	CDKN2A	883	63	47	3	-0.161	0.574	0.574	Mutual exclusivity
RB1	CCND1	777	62	153	4	-1.61	0.013	0.13	Mutual exclusivity
RB1	CDK4	916	66	14	0	<-3	0.38	0.573	Mutual exclusivity
RB1	CDK6	920	66	10	0	<-3	0.502	0.573	Mutual exclusivity
CDKN2A	CCND1	794	45	152	5	-0.785	0.172	0.431	Mutual exclusivity
CDKN2A	CDK4	933	49	13	1	0.551	0.516	0.573	Co-occurrence
CDKN2A	CDK6	937	49	9	1	1.087	0.404	0.573	Co-occurrence
CCND1	CDK4	830	152	9	5	1.601	0.055	0.275	Co-occurrence
CCND1	CDK6	830	156	9	1	-0.758	0.516	0.573	Mutual exclusivity
CDK4	CDK6	973	13	9	1	>3	0.133	0.431	Co-occurrence

HNSC

A	B	Neither	A Not B	B Not A	Both	Log2 Odds Ratio	p-Value	q-Value	Tendency
RB1	CDKN2A	226	16	248	6	-1.549	0.018	0.09	Mutual exclusivity
RB1	CCND1	358	20	116	2	-1.696	0.073	0.145	Mutual exclusivity
RB1	CDK4	467	21	7	1	1.668	0.306	0.437	Co-occurrence
RB1	CDK6	452	21	22	1	-0.032	0.729	0.729	Mutual exclusivity
CDKN2A	CCND1	206	172	36	82	1.448	<0.001	**<0.001**	**Co-occurrence**
CDKN2A	CDK4	236	252	6	2	-1.68	0.127	0.212	Mutual exclusivity
CDKN2A	CDK6	231	242	11	12	0.058	0.548	0.671	Co-occurrence
CCND1	CDK4	372	116	6	2	0.096	0.604	0.671	Co-occurrence
CCND1	CDK6	364	109	14	9	1.102	0.069	0.145	Co-occurrence
CDK4	CDK6	467	6	21	2	2.89	0.049	0.145	Co-occurrence

Supplementary Figure 3: Analysis of mutual exclusive or co-occurring relationships in representative tumor types that exhibit frequent RB1 loss/mutation. The log-odds ratio and associated p-value are summarized.

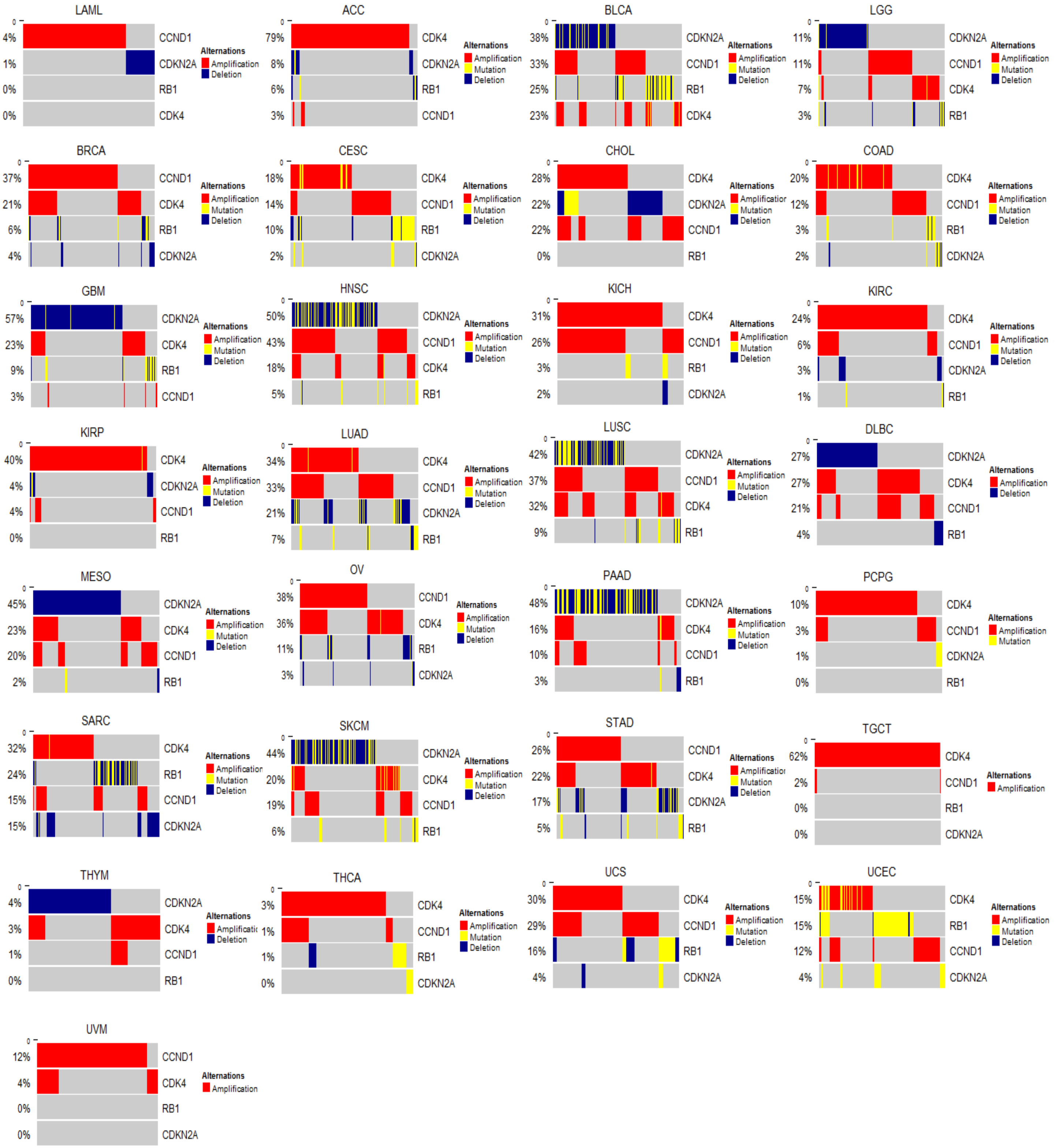

Supplementary Figure 4: Oncoprints of cancer types exhibiting mutation (yellow), homozygous deletion (blue) and amplification (red) of the indicated genes. Gene are organized by frequency of alteration indicating the disparate dominant mechanisms of RB-pathway deregulation across tumor types.

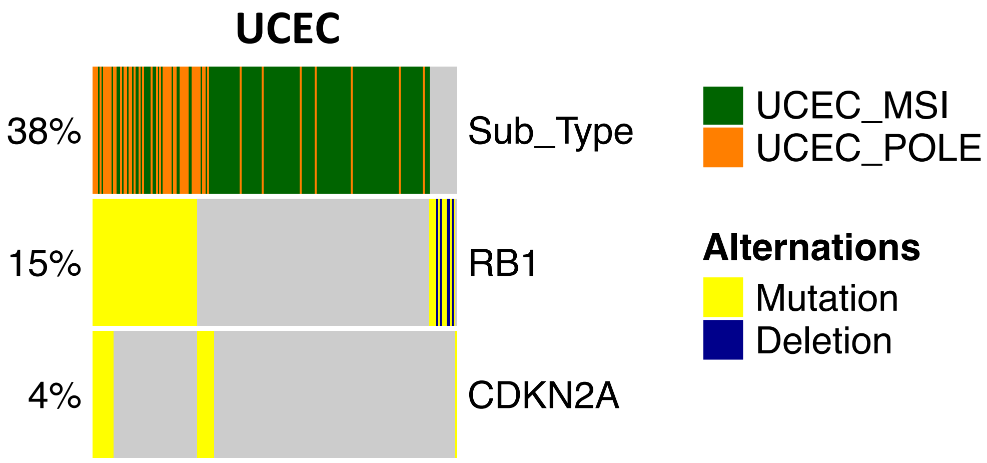

Supplementary Figure 5: Oncoprint shows the relationship of RB1 and CDKN2A genetic alterations vs. MSI or POLE mutation status in UCEC. The co-occurring alterations in RB1 and CDKN2A are largely associated with these mutator phenotypes. The percentages indicate the frequency of the observed genetic alterations in the cohort.

Figure S6

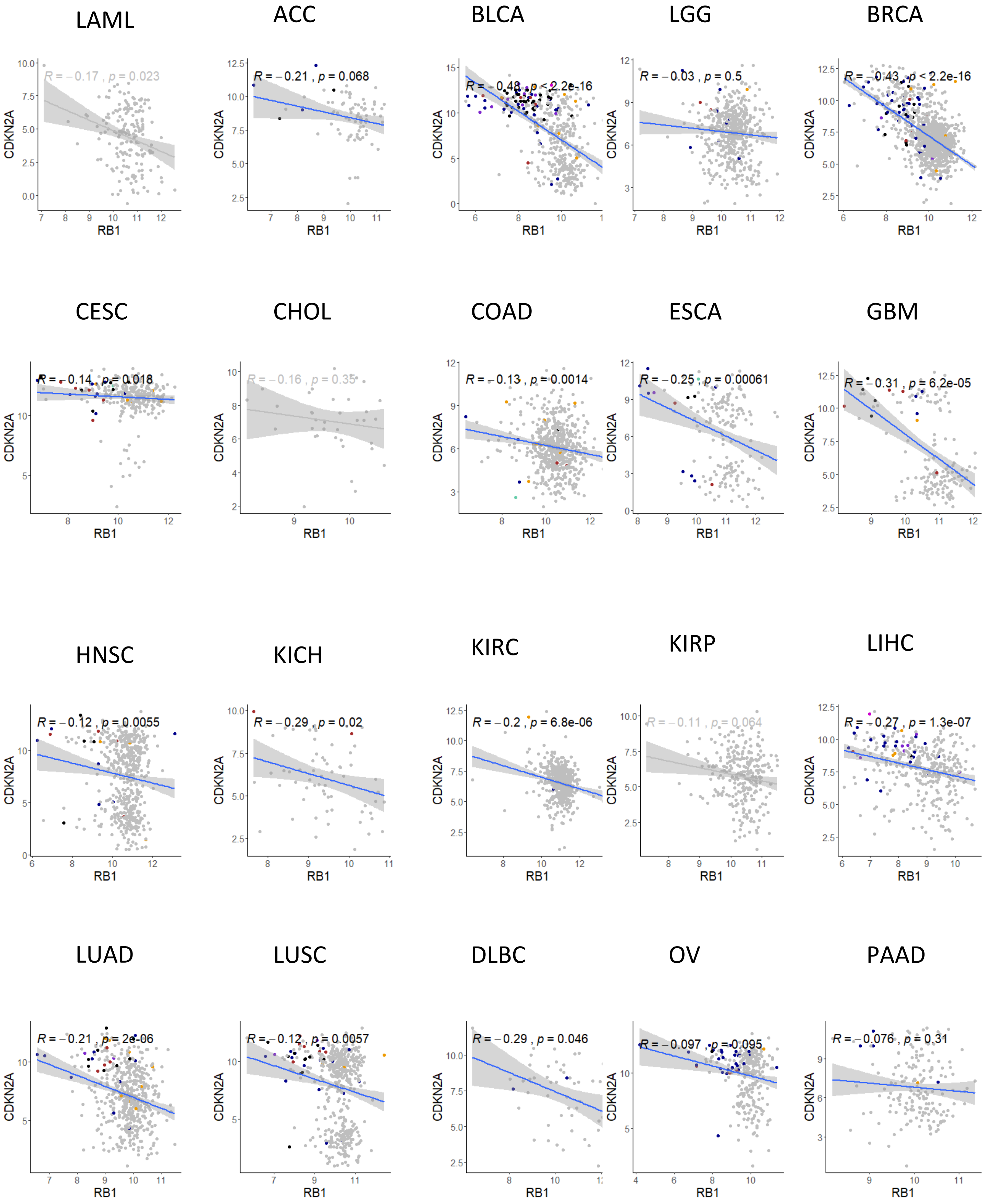

LAML
R = −0.17 , p = 0.023
ACC
R = −0.21 , p = 0.068
BLCA
R = −0.48 , p < 2.2e-16
LGG
R = −0.03 , p = 0.5
BRCA
R = −0.43 , p < 2.2e-16
CESC
R = −0.14 , p = 0.018
CHOL
R = −0.16 , p = 0.35
COAD
R = −0.13 , p = 0.0014
ESCA
R = −0.25 , p = 0.00061
GBM
R = −0.31 , p = 6.2e-05
HNSC
R = −0.12 , p = 0.0055
KICH
R = −0.29 , p = 0.02
KIRC
R = −0.2 , p = 6.8e-06
KIRP
R = −0.11 , p = 0.064
LIHC
R = −0.27 , p = 1.3e-07
LUAD
R = −0.21 , p = 2e-06
LUSC
R = −0.12 , p = 0.0057
DLBC
R = −0.29 , p = 0.046
OV
R = −0.097 , p = 0.095
PAAD
R = −0.076 , p = 0.31
CDKN2A
RB1

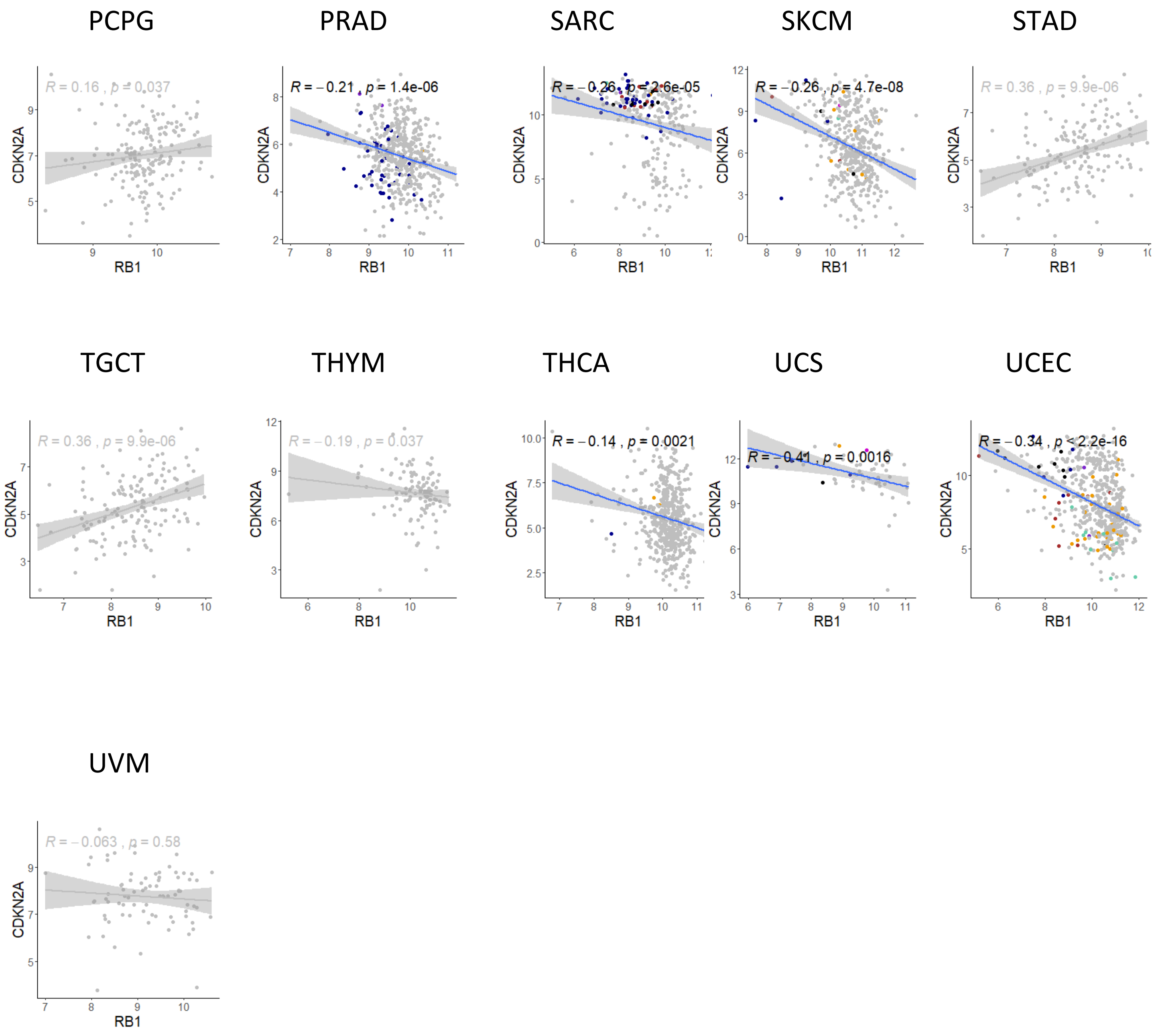

Supplementary Figure 6: Pearson correlation of the relationship between CDKN2A and RB1 gene expression across all tumors in the TCGA pan-cancer data sets (R-value and related p-value are shown). The presence of different mutations in RB1 are shown in the legend, the shading denotes the 95% confidence interval.

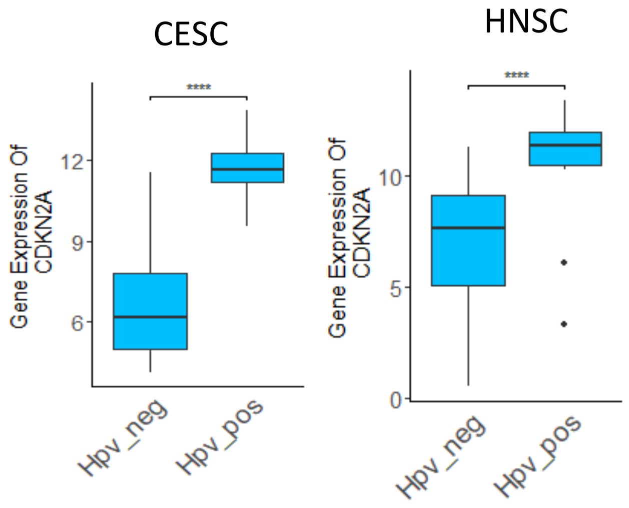

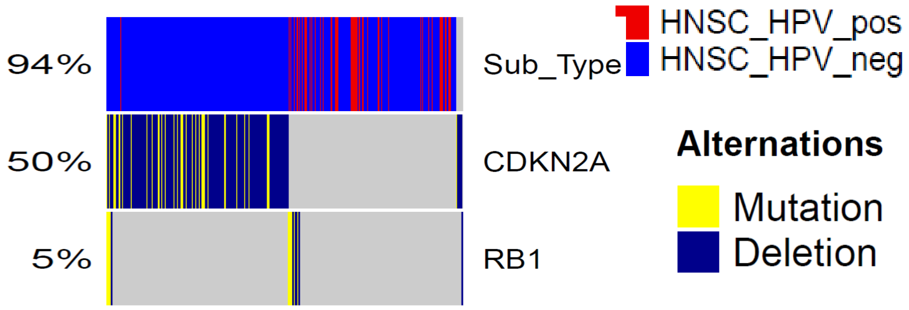

Supplementary Figure 7: Stratification of the expression of CDKN2A by the HPV-status in HNSC and CESC cases. For HNSC cases 72 are positive and 415 are negative for HPV. For CESC cases 169 are positive and 9 are negative for HPV. The percentages indicate the frequency of the observed genetic alterations in the cohort.

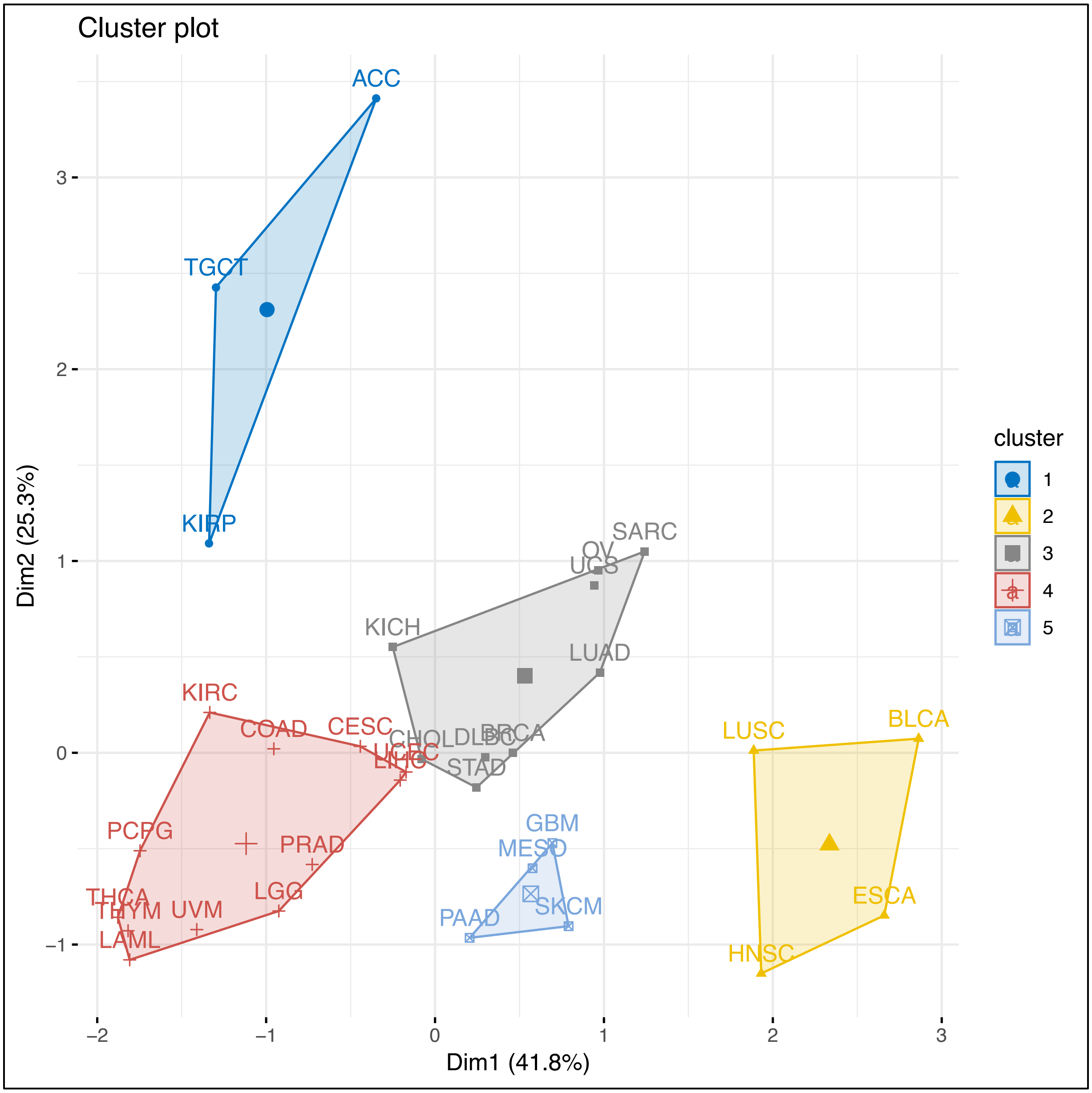

Supplementary Figure 8: Principle component analysis of all samples and each of the tumor types based on the frequency of CDKN2A, CDK4, CCND1, and RB1 genetic alterations. K-Means clustering yields five discrete clusters of tumor types.

Supplementary Figure 9

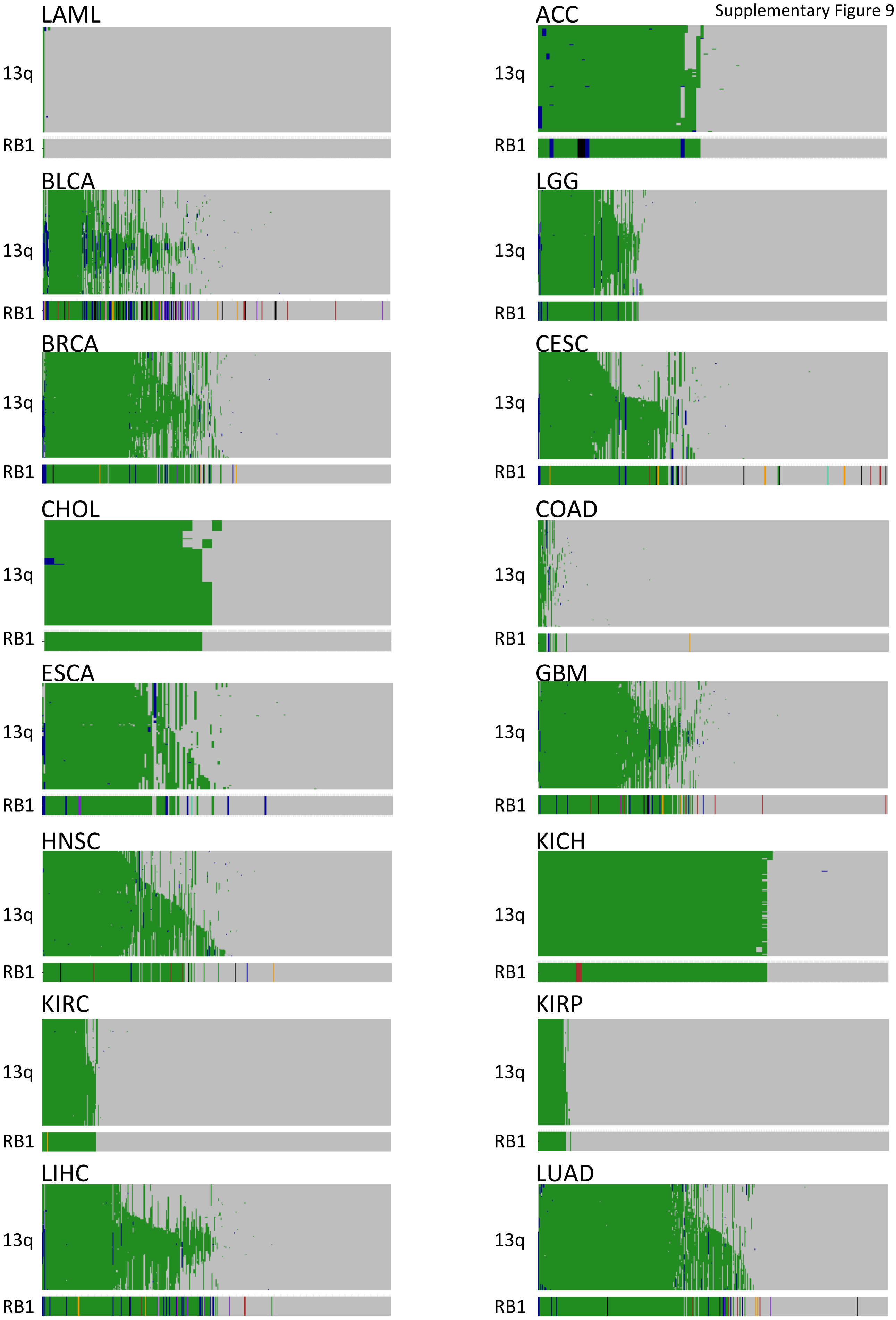

Figure S9

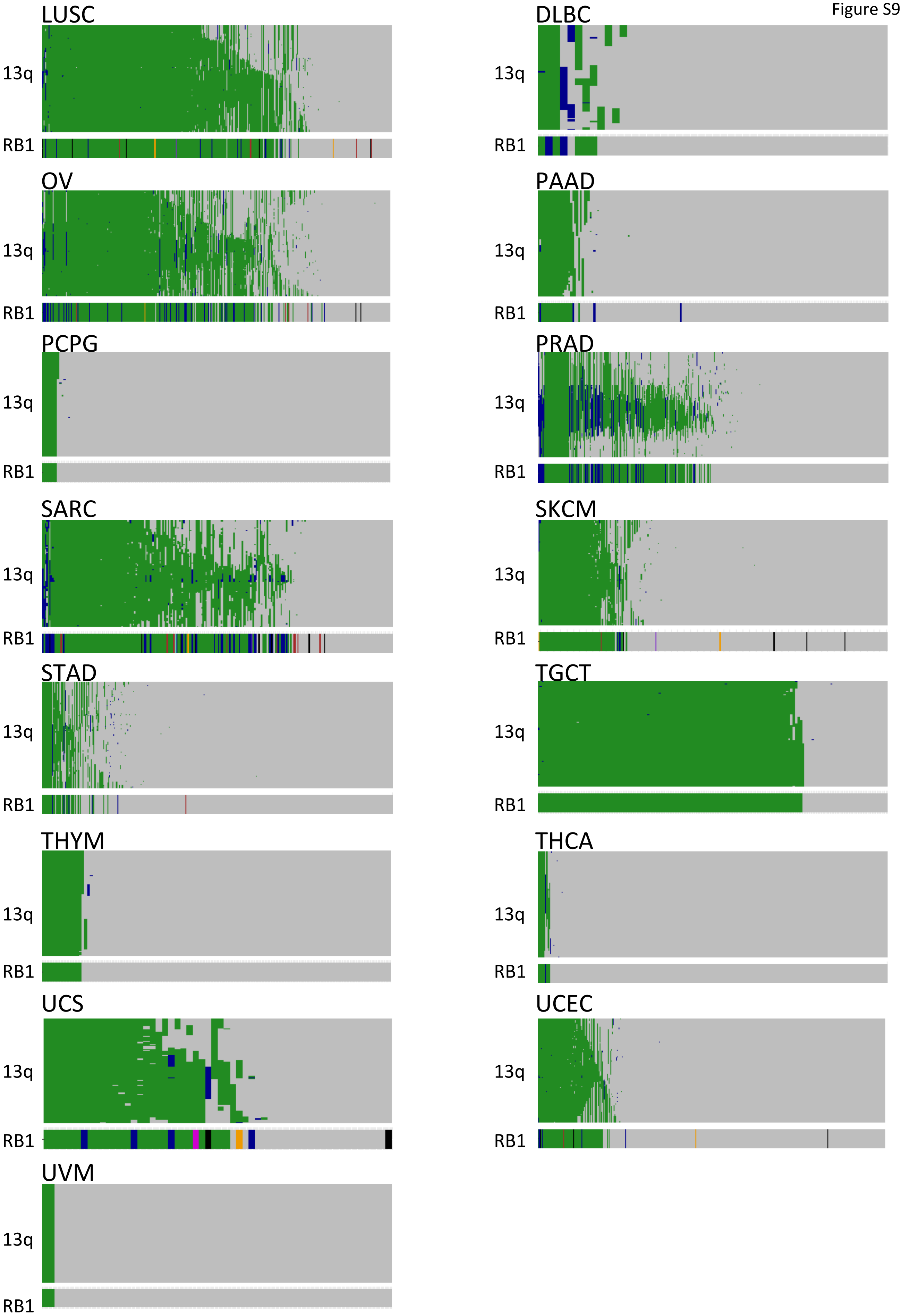
LUSC
13q
RB1
DLBC
13q
RB1
OV
13q
RB1
PAAD
13q
RB1
PCPG
13q
RB1
PRAD
13q
RB1
SARC
13q
RB1
SKCM
13q
RB1
STAD
13q
RB1
TGCT
13q
RB1
THYM
13q
RB1
THCA
13q
RB1
UCS
13q
RB1
UCEC
13q
RB1
UVM
13q
RB1

Supplementary Figure 9: Histograms summarize the copy number analysis of single copy loss (green) and deletion (blue) of genes in *cis* along chromosome arm 13q. A total of 1057 genes are illustrated. The behavior of RB1 is shown in the histogram below, all cases for all tumor types are shown. Legend denotes different forms of RB1 gene alterations beyond copy number changes

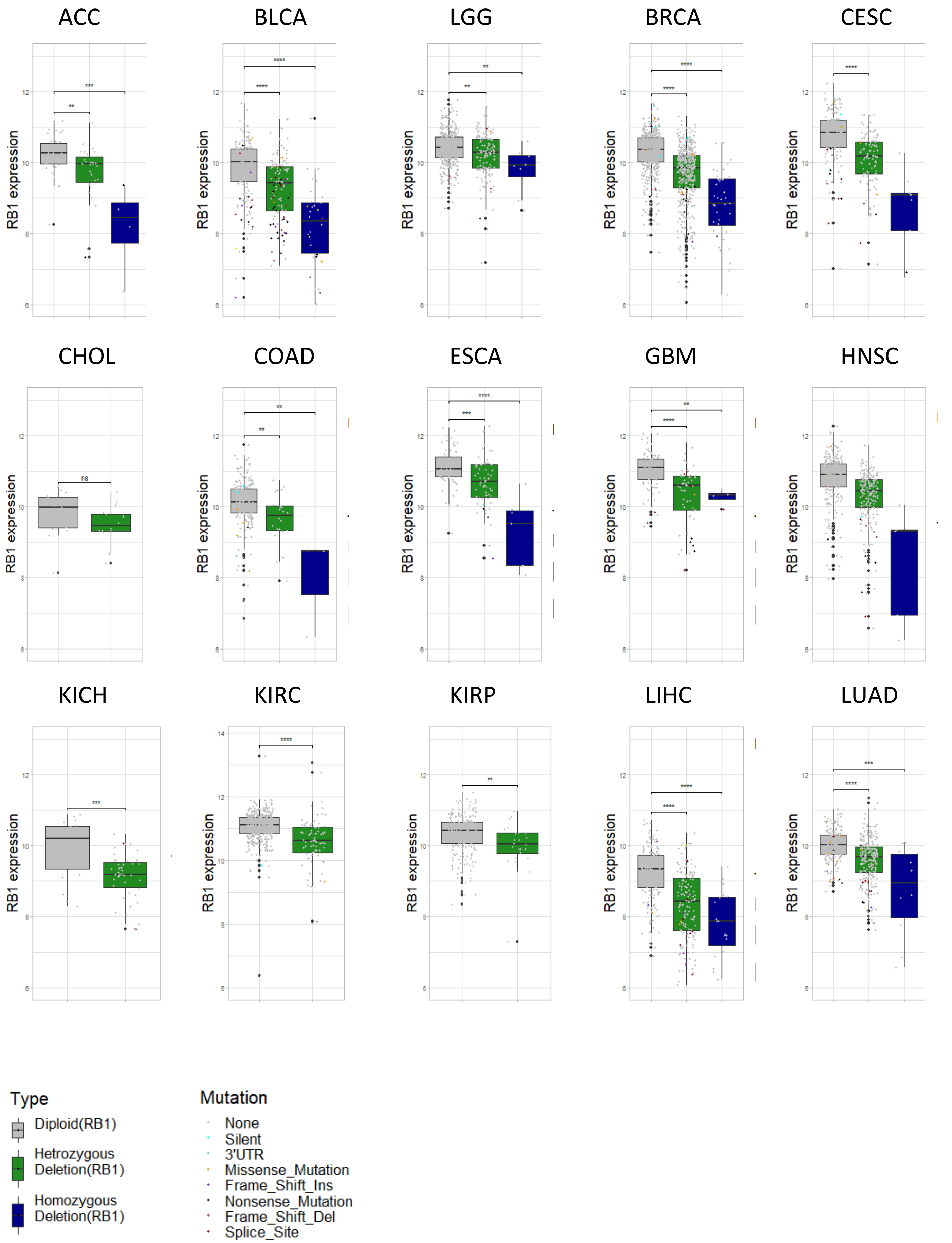
ACC
BLCA
LGG
BRCA
CESC
CHOL
COAD
ESCA
GBM
HNSC
KICH
KIRC
KIRP
LIHC
LUAD
RB1 expression
ns
Type
Diploid(RB1)
Hetrozygous Deletion(RB1)
Homozygous Deletion(RB1)
Mutation
None
Silent
3'UTR
Missense_Mutation
Frame_Shift_Ins
Nonsense_Mutation
Frame_Shift_Del
Splice_Site

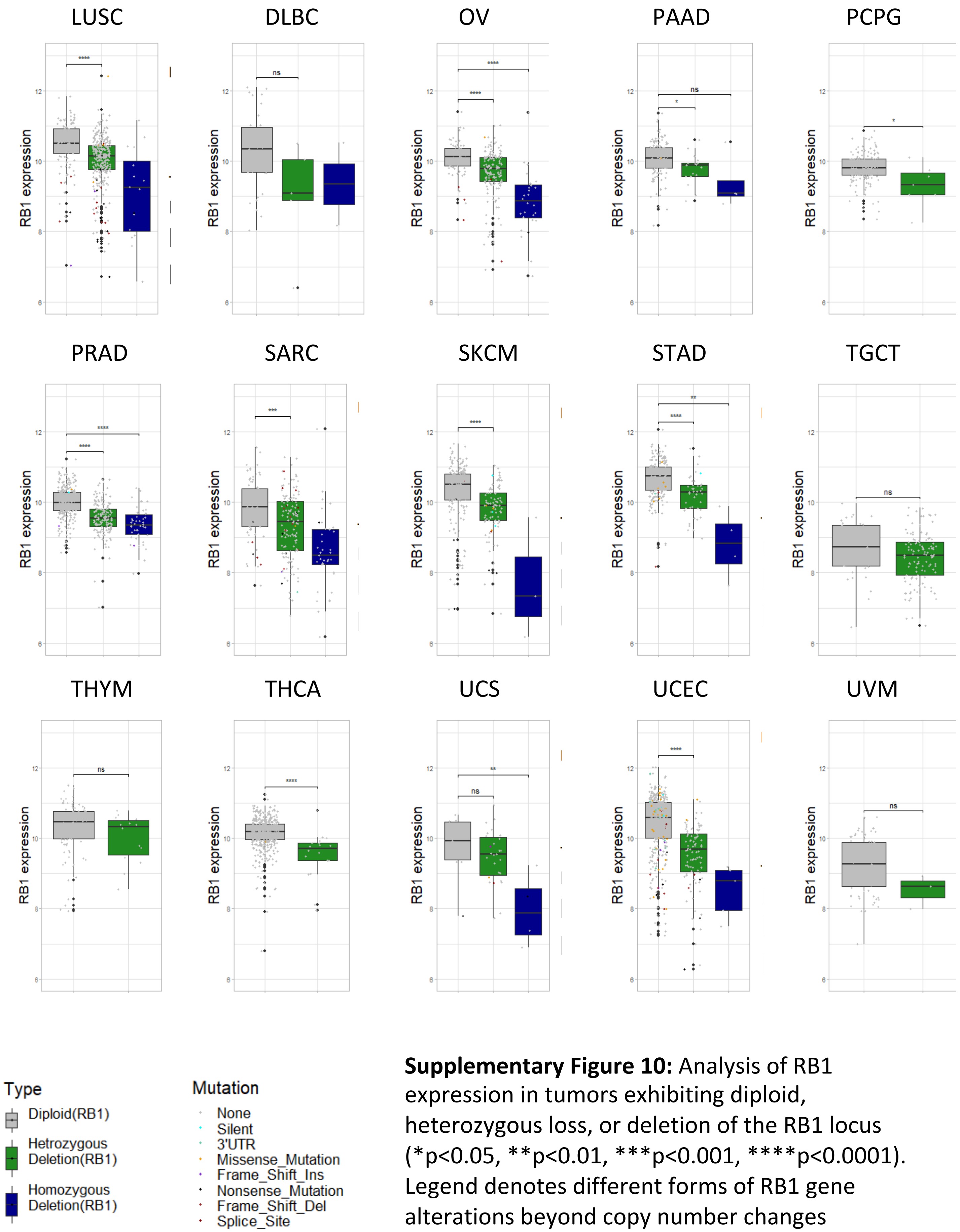

Supplementary Figure 10: Analysis of RB1 expression in tumors exhibiting diploid, heterozygous loss, or deletion of the RB1 locus (*p<0.05, **p<0.01, ***p<0.001, ****p<0.0001). Legend denotes different forms of RB1 gene alterations beyond copy number changes

Supplementary Figure 11

ACC

Disease Free Survival

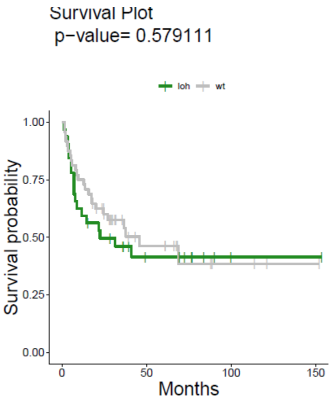

Overall Survival

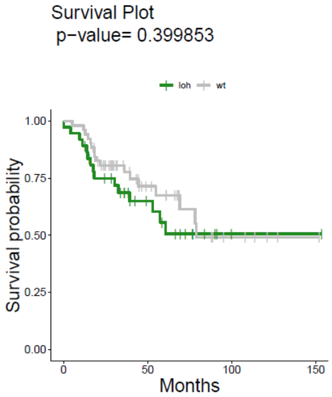

BLCA

Disease Free Survival

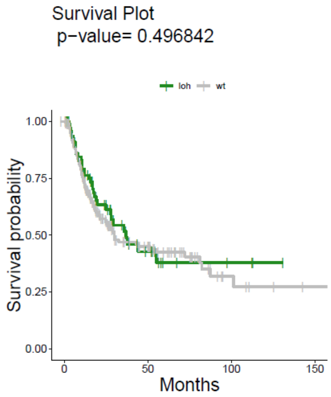

Overall Survival

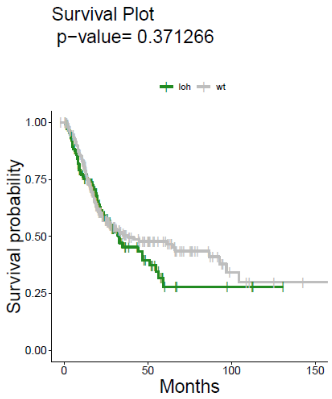

LGG

Disease Free Survival

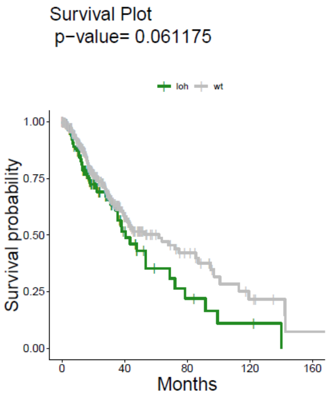

Overall Survival

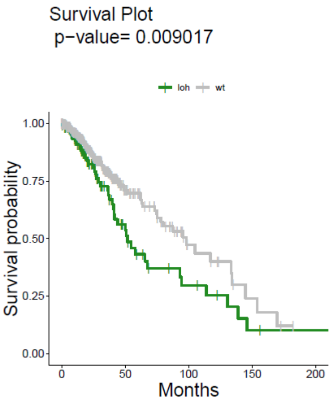

BRCA

Disease Free Survival

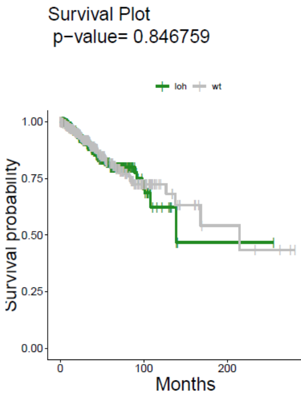

Overall Survival

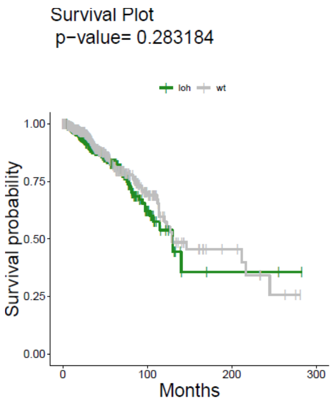

CESC

Disease Free Survival

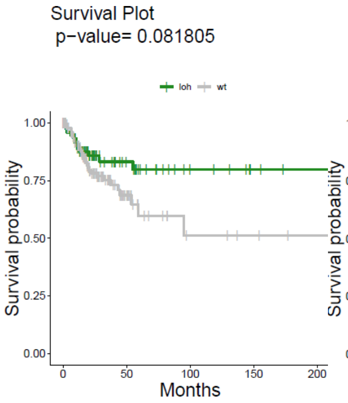

Overall Survival

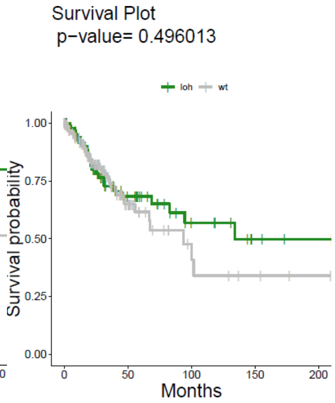

CHOL

Disease Free Survival

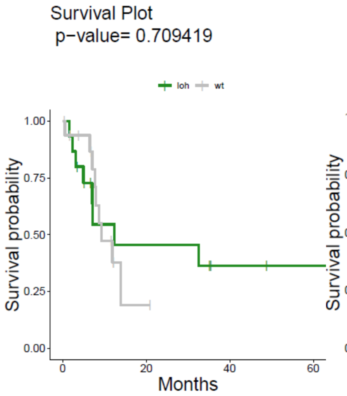

Overall Survival

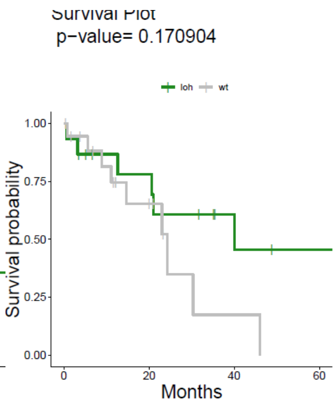

Supplementary Figure 11

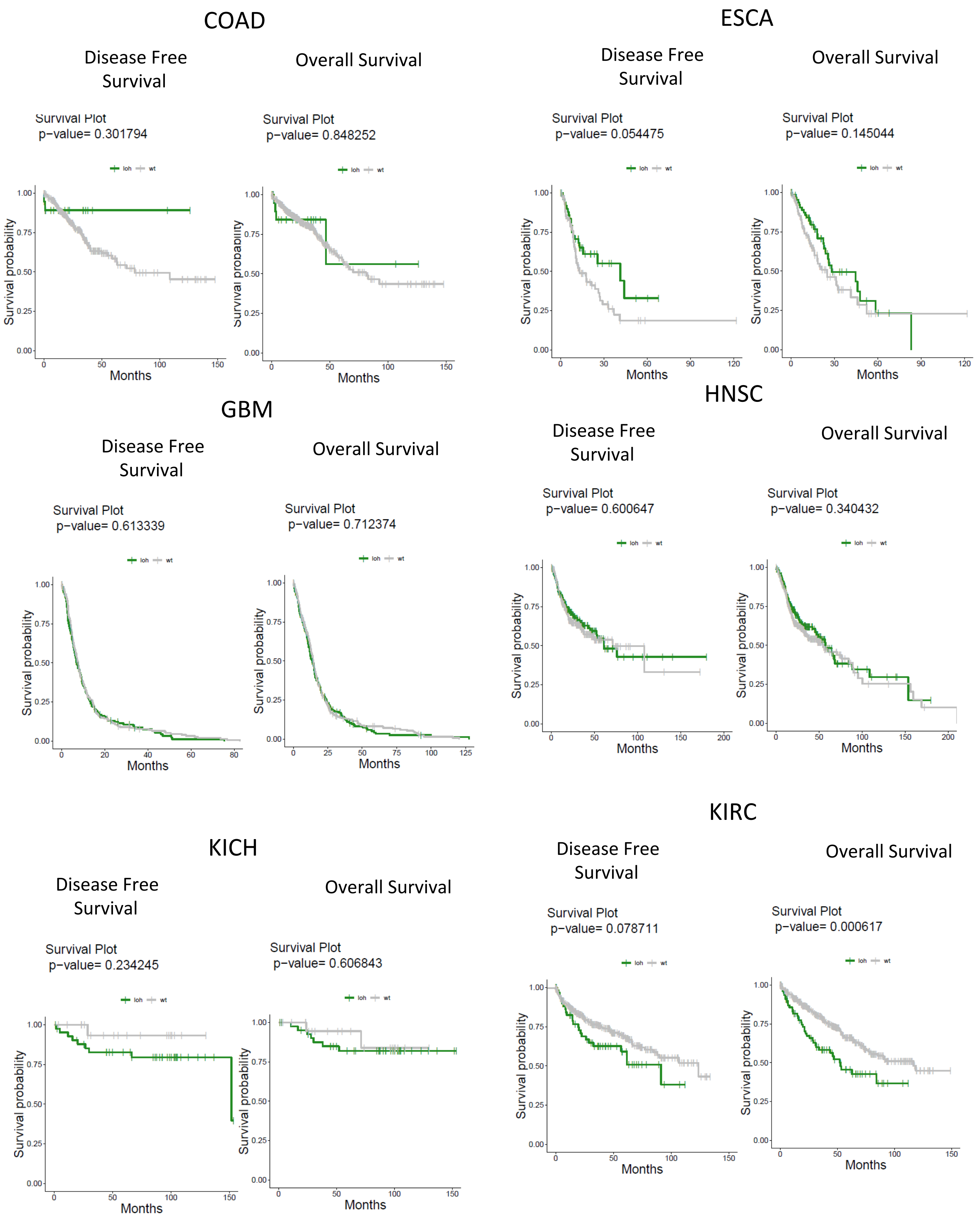

Supplementary Figure 11

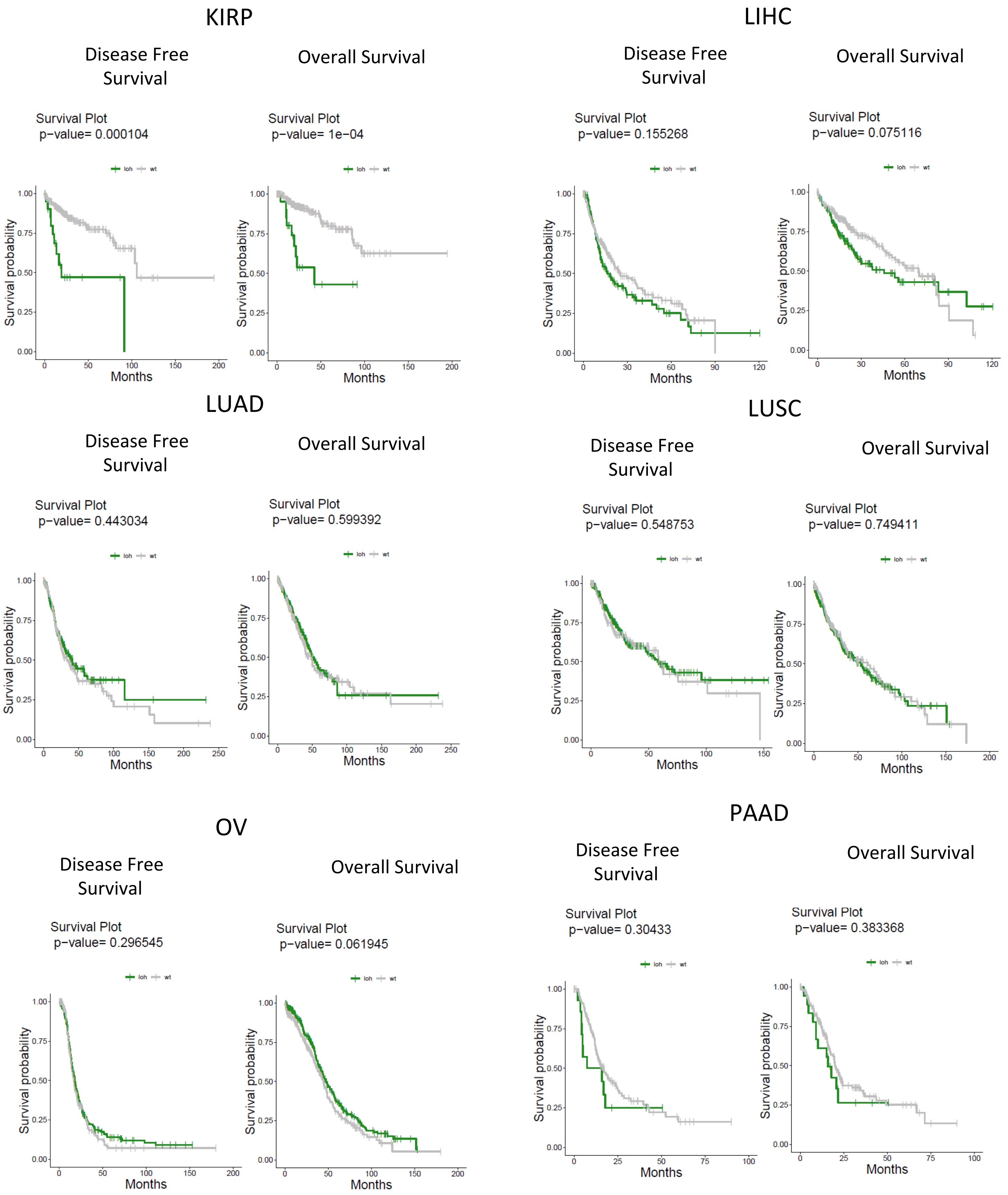

Supplementary Figure 11

PRAD

Disease Free Survival

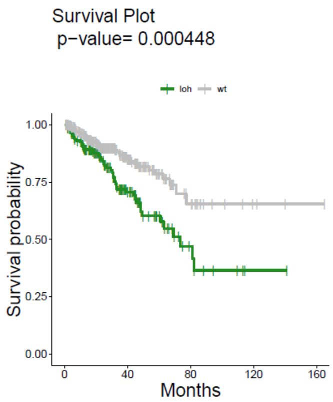

Overall Survival

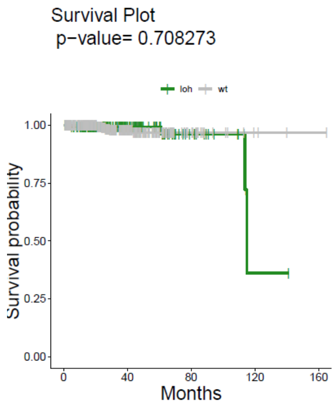

SARC

Disease Free Survival

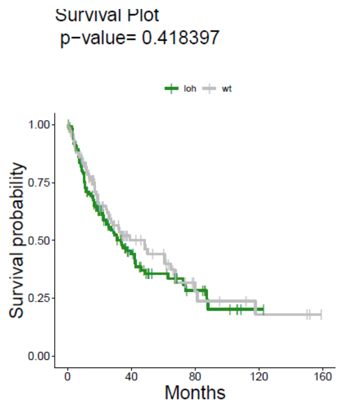

Overall Survival

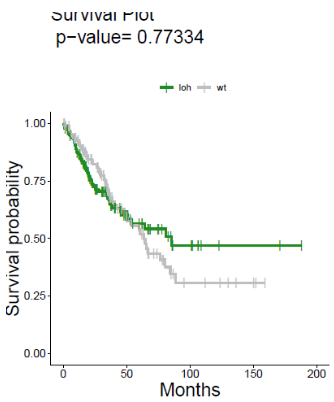

SKCM

Disease Free Survival

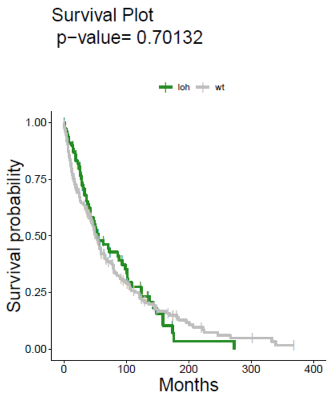

Overall Survival

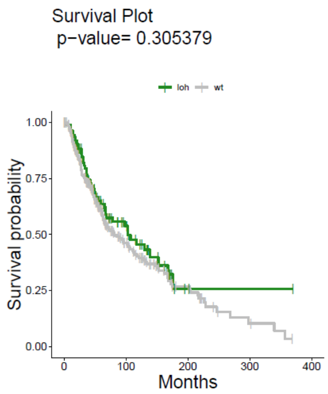

STAD

Disease Free Survival

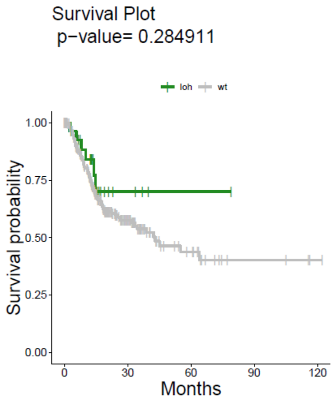

Overall Survival

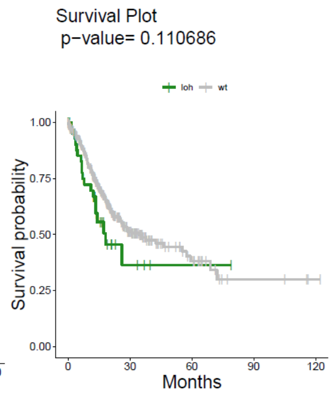

TGCT

Disease Free Survival

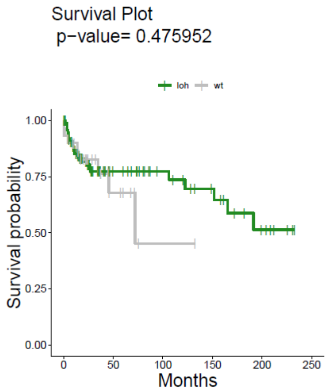

Overall Survival

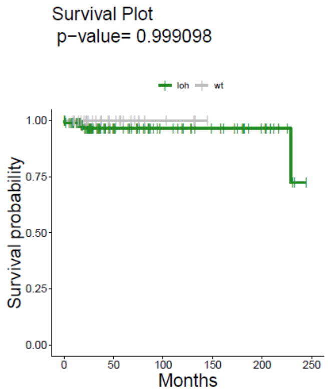

THYM

Disease Free Survival

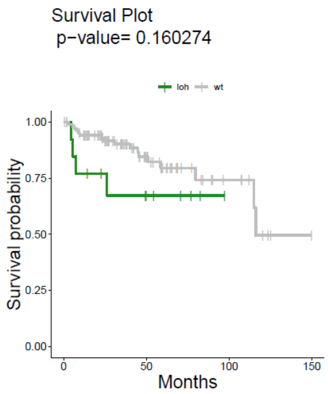

Overall Survival

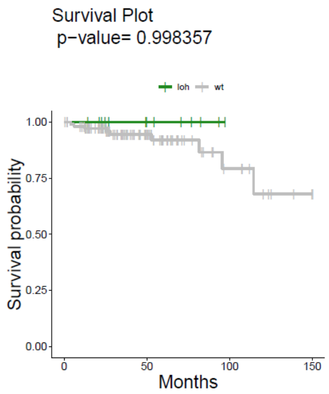

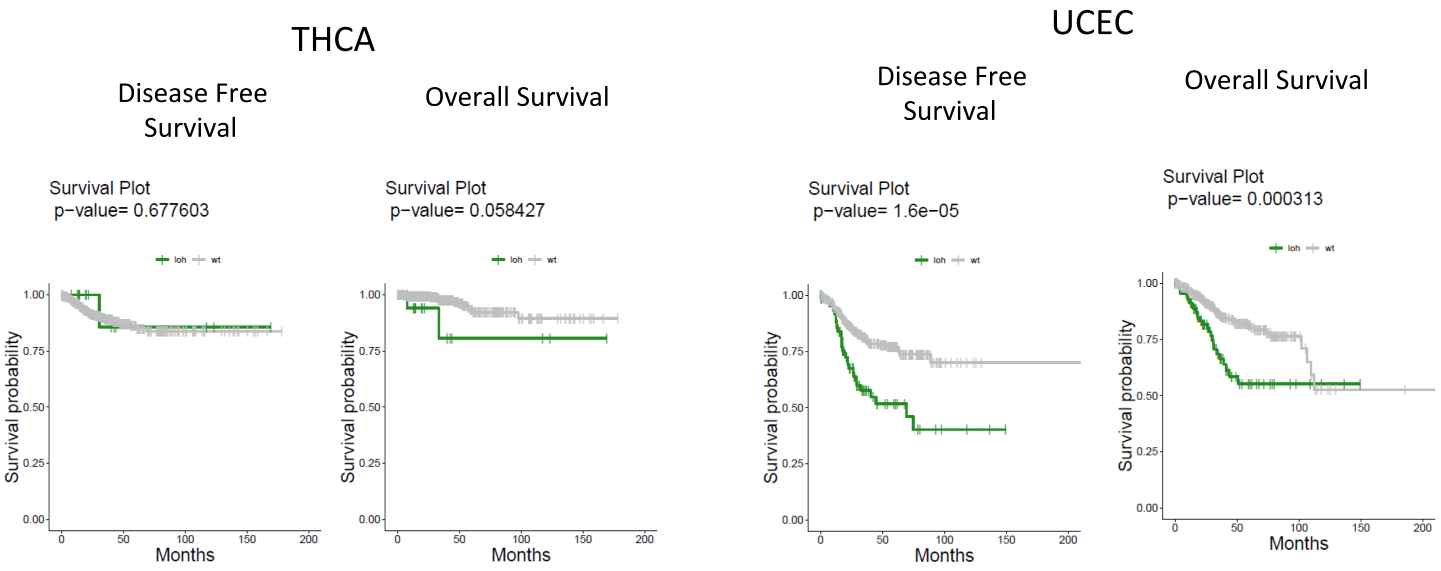

Supplementary Figure 11: Kaplan-Meier analysis of pan-cancer tumor types stratified by diploid vs. heterozygous loss of RB1 locus. Statistical significance was determined by log-rank test. Both the disease-free survival and overall survival results are shown for all cases for which the data is available. NA indicates the survival data is not available for that tumor type.

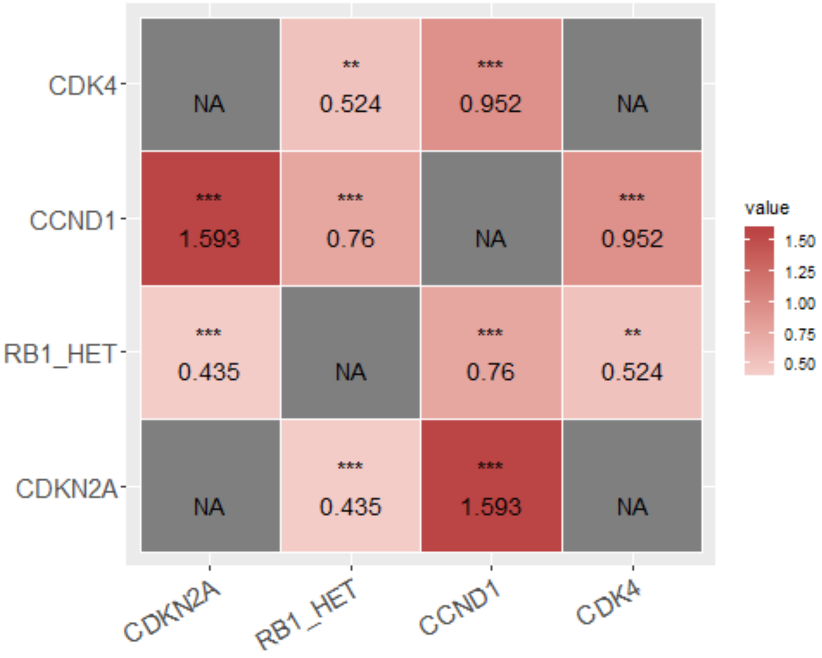

Supplementary Figure 12: Matrix plot shows the Odds-ratio for co-occurrence between RB1 heterozygosity and other RB-pathway alterations across all tumor types in the TCGA pan-cancer. Statistical significance is shown (**p<0.01, ***p<0.001).

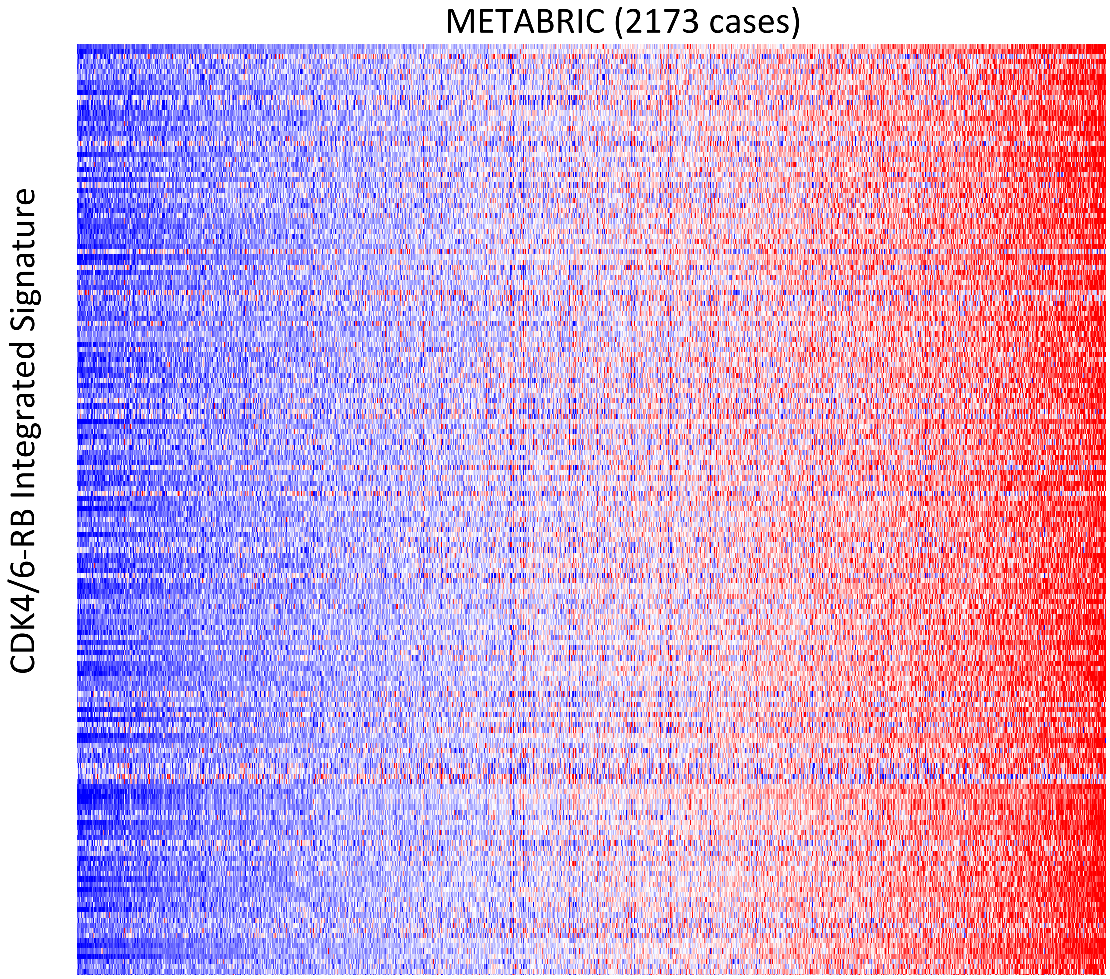

Supplementary Figure 13: Heatmap shows the expression of the 182 genes of CDK4/6-RB integrated signature clustered by average signature value. The clinical cases are from the METABRIC data set of 2173 breast cancer samples.

Supplementary Figure 14

LAML

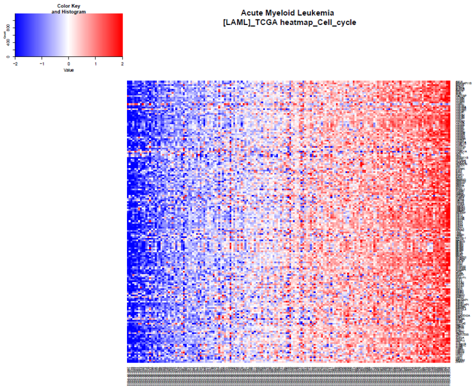

Disease Free Survial

NA

Overall Survival

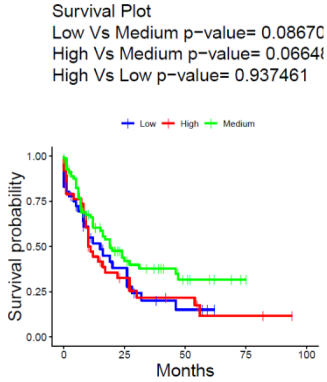

ACC

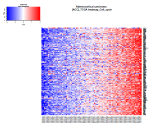

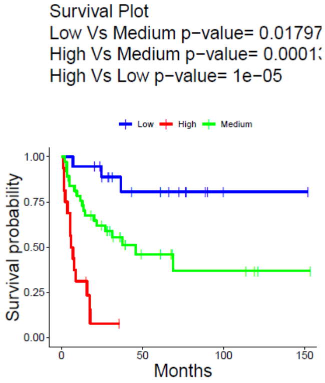

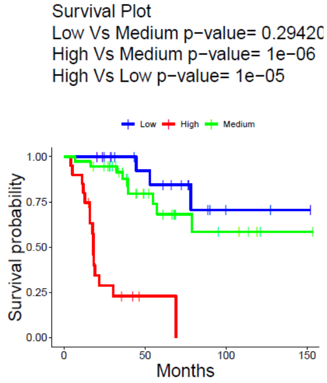

BLCA

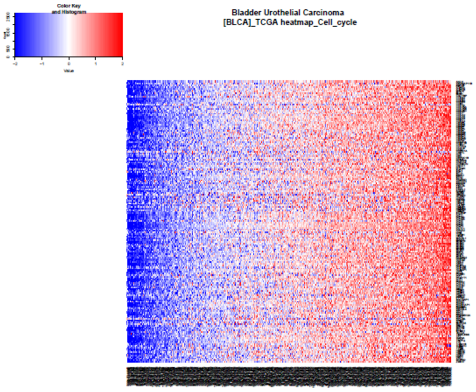

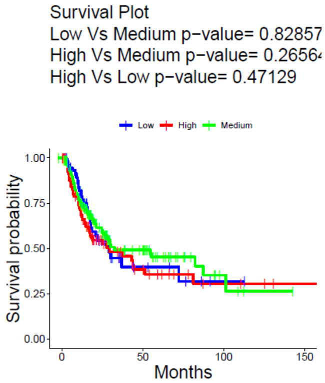

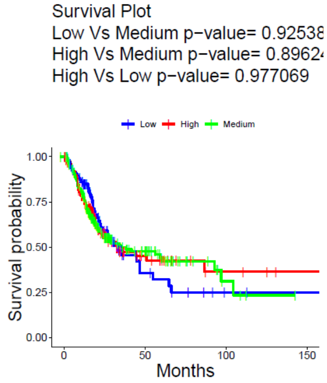

LGG

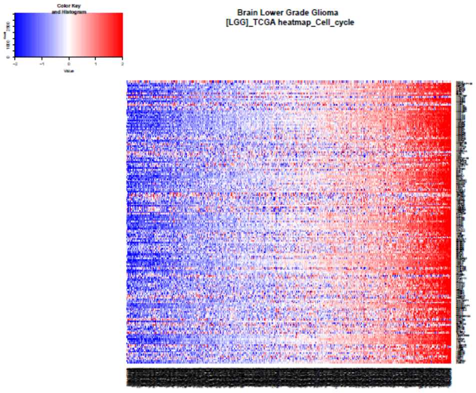

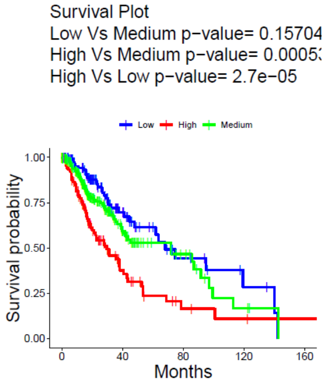

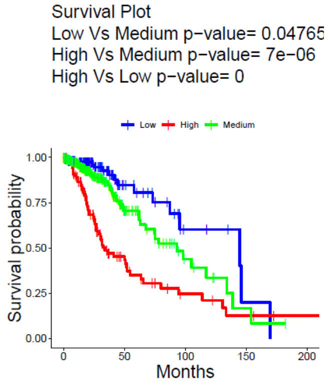

Supplementary Figure 14

Disease Free Survial | Overall Survival

BRCA

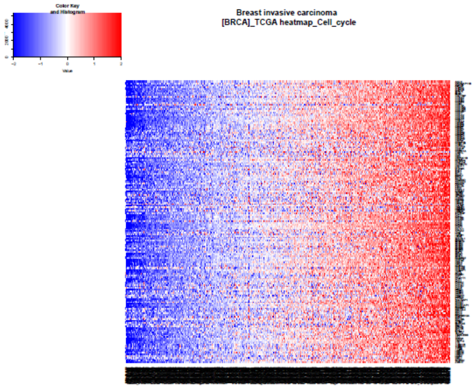

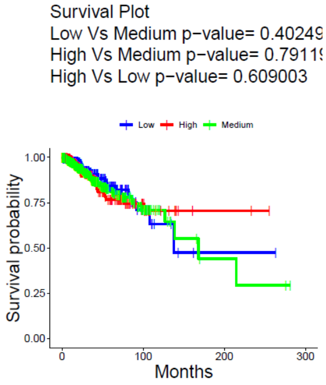

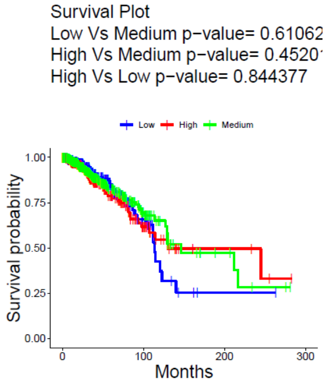

CESC

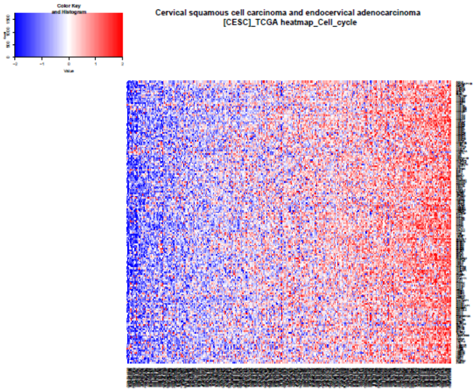

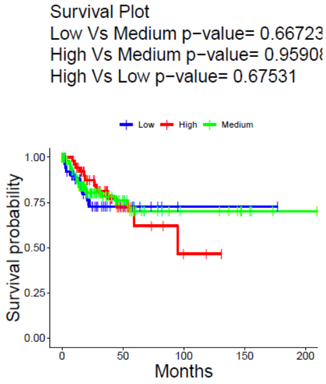

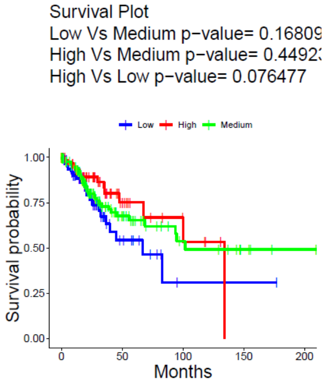

CHOL

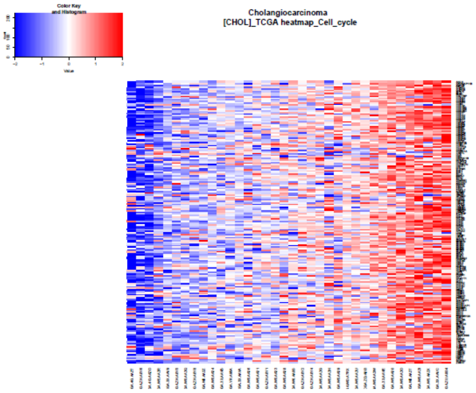

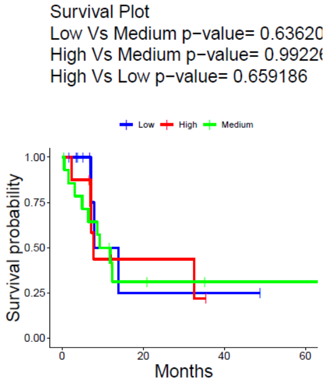

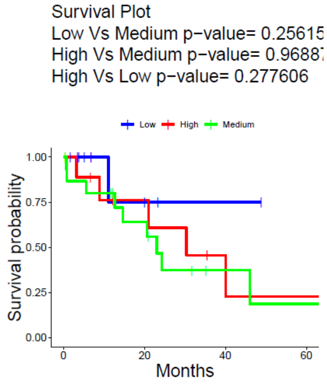

COAD

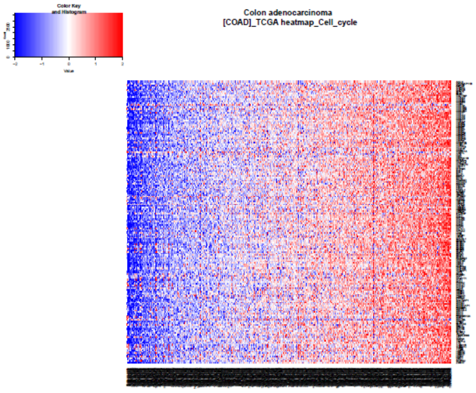

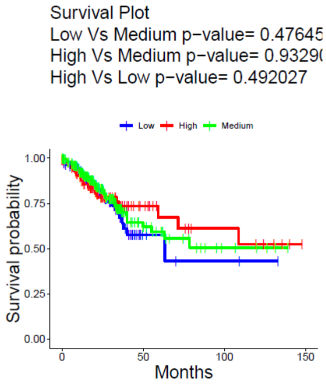

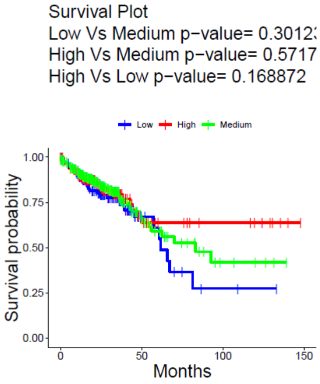

Supplementary Figure 14

Disease Free Survial

Overall Survival

ESCA

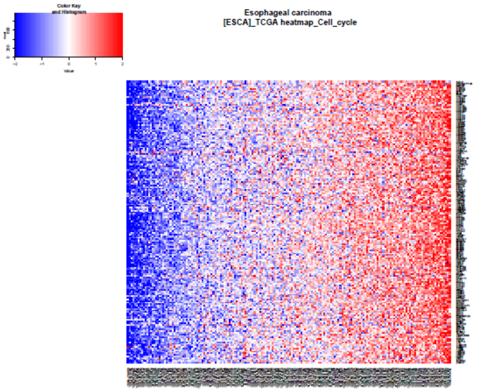

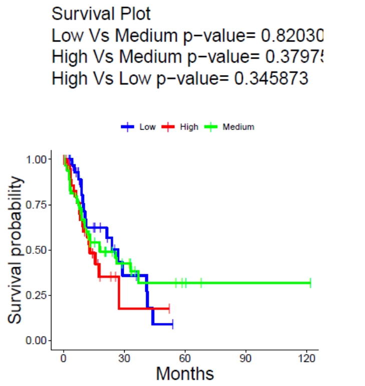

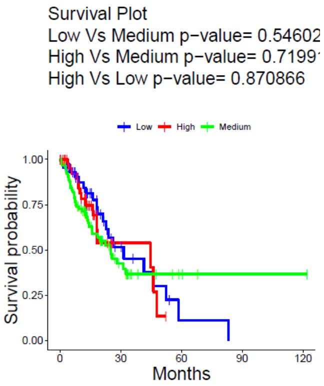

GBM

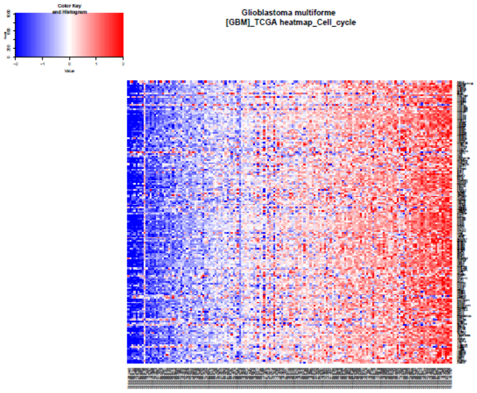

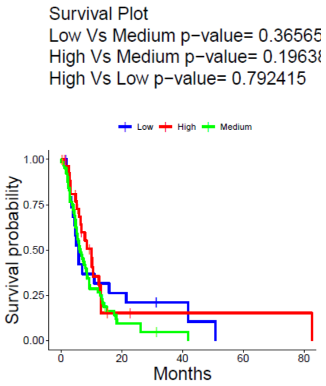

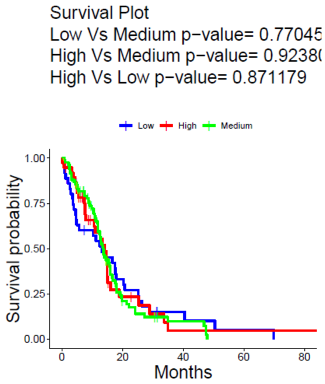

HNSC

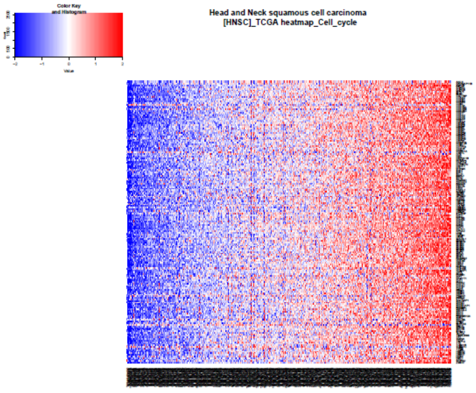

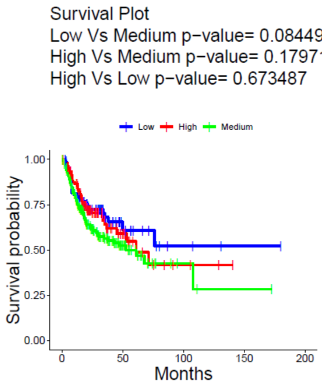

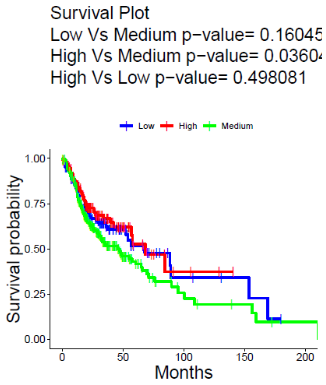

KICH

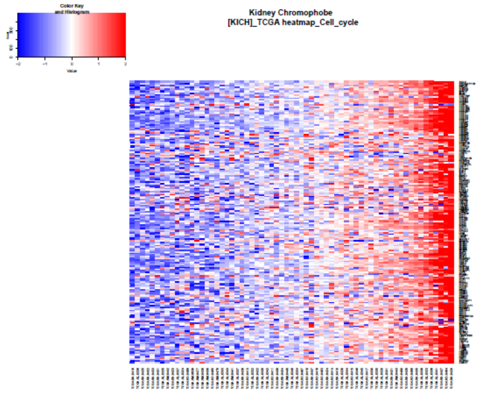

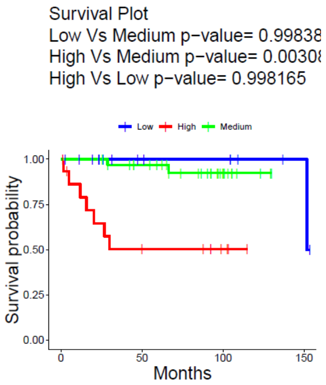

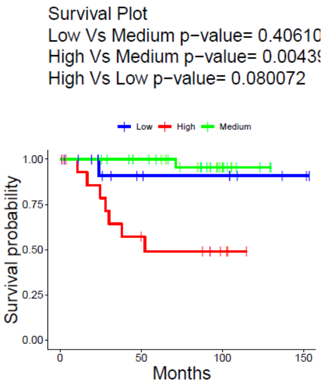

Supplementary Figure 14

Disease Free Survial

Overall Survival

KIRC

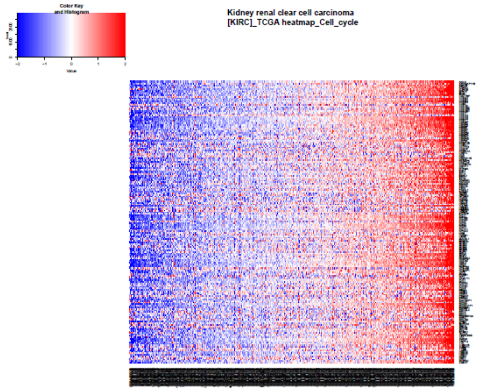

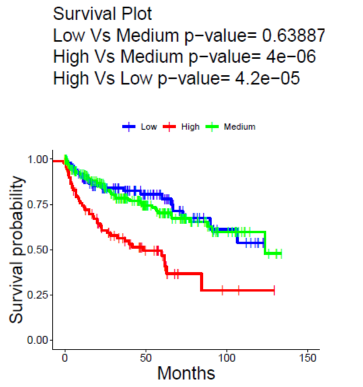

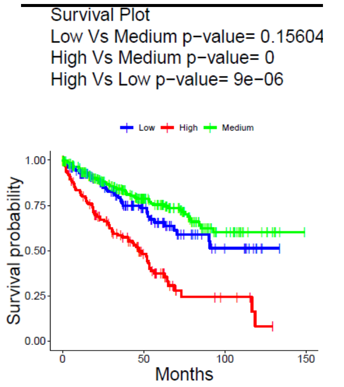

KIRP

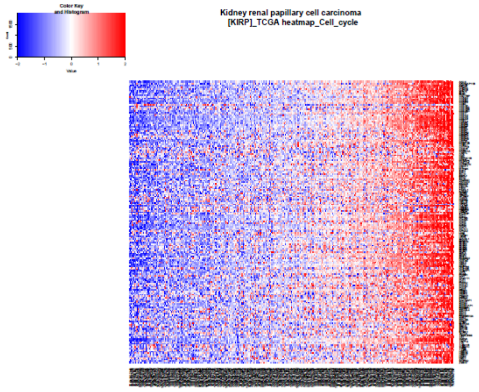

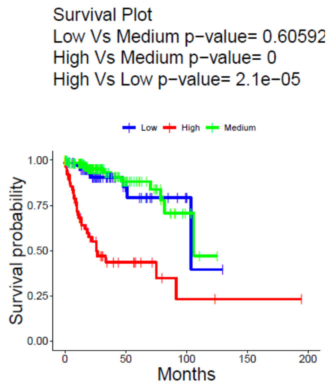

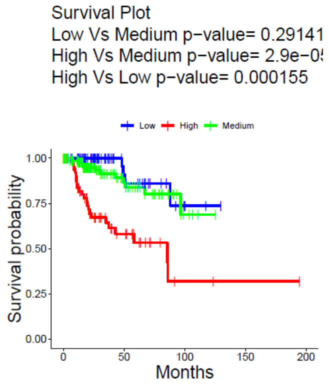

LIHC

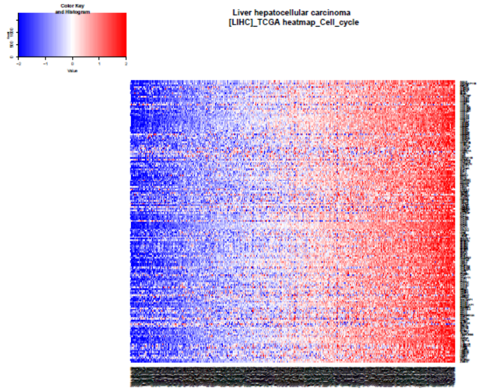

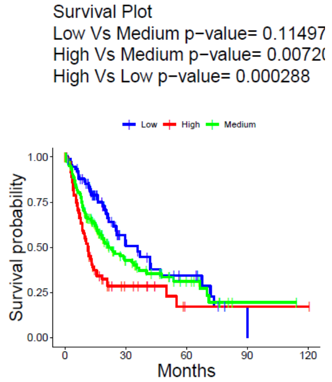

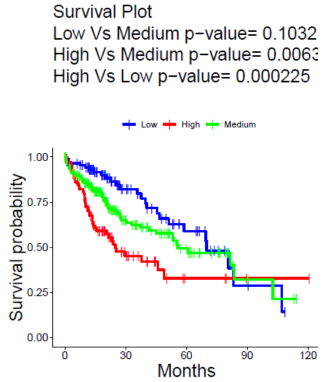

LUAD

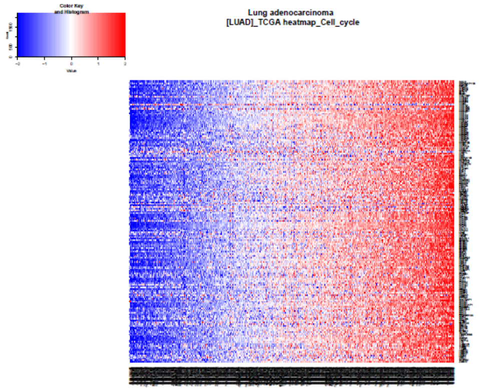

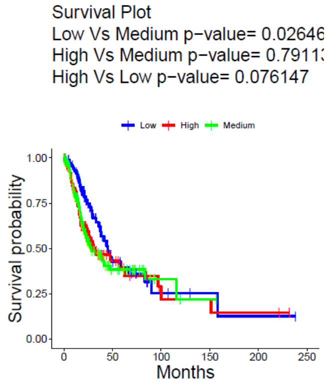

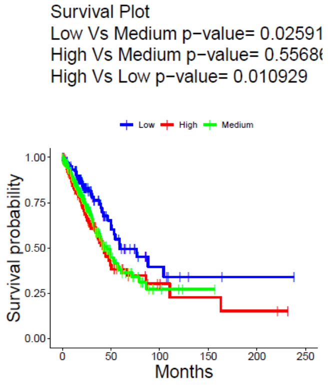

Supplementary Figure 14

LUSC

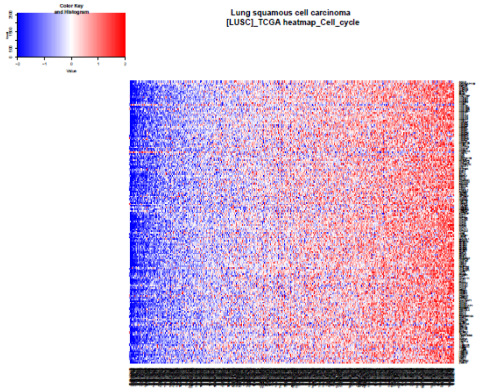

Disease Free Survial

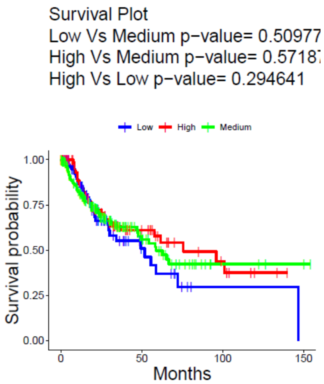

Overall Survival

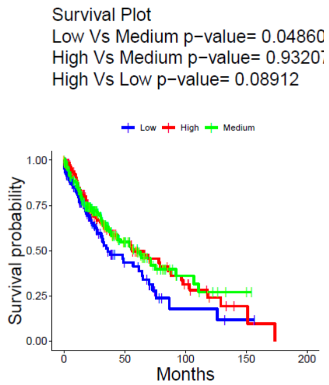

DLBC

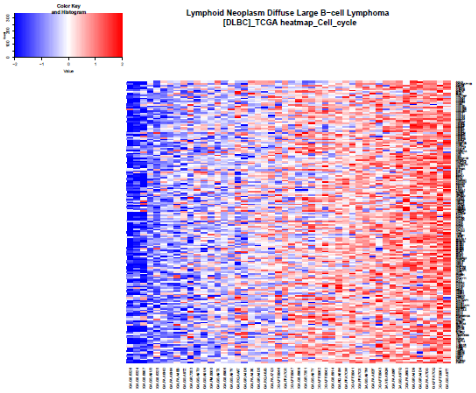

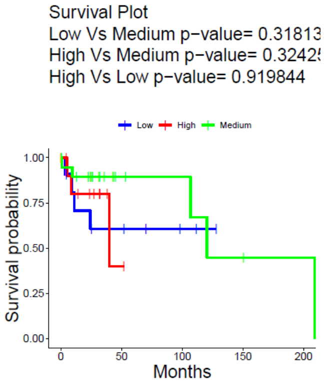

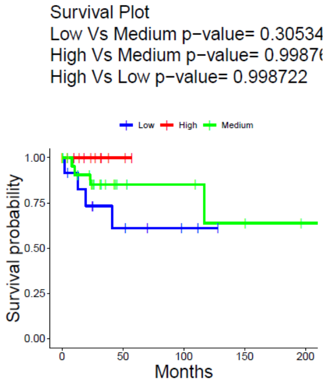

OV

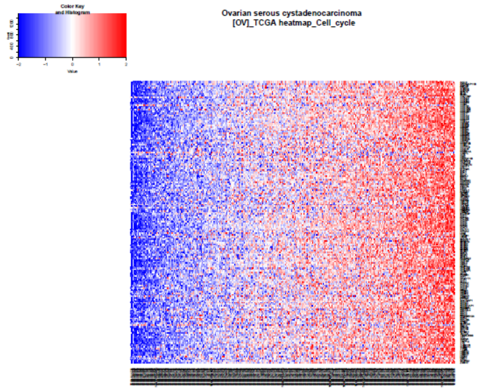

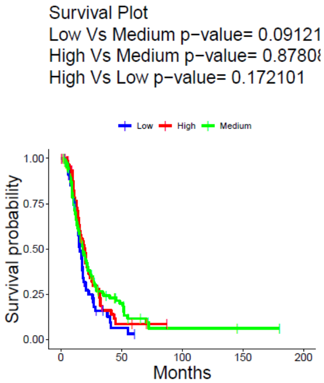

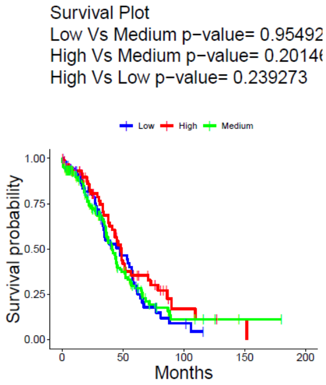

PAAD

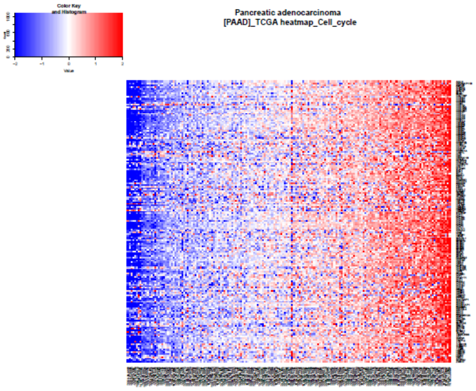

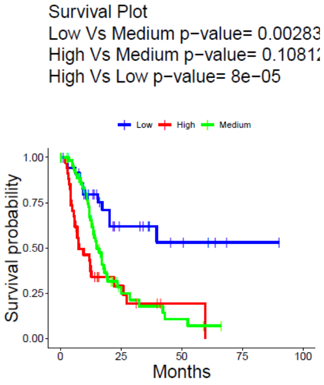

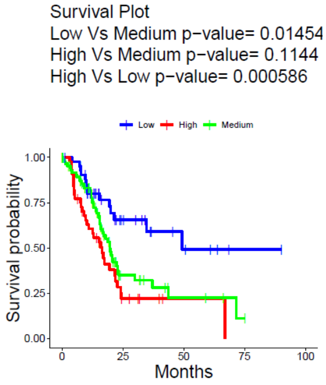

Supplementary Figure 14

	Disease Free Survial	Overall Survival

PCPG

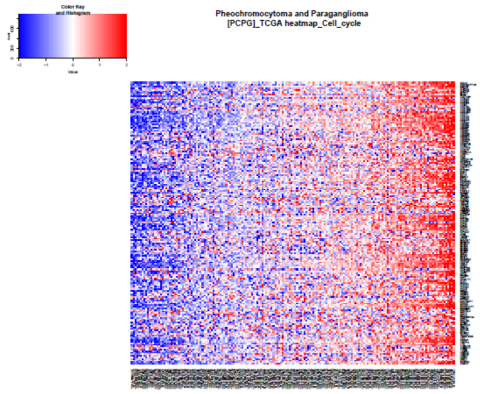

NA

NA

PRAD

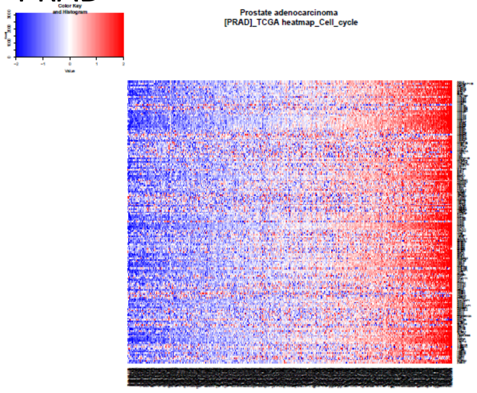

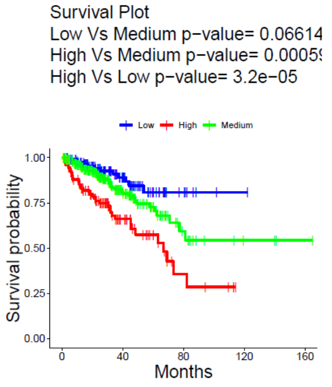

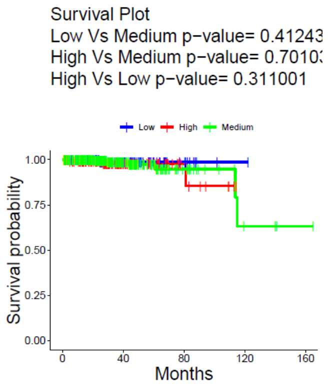

SARC

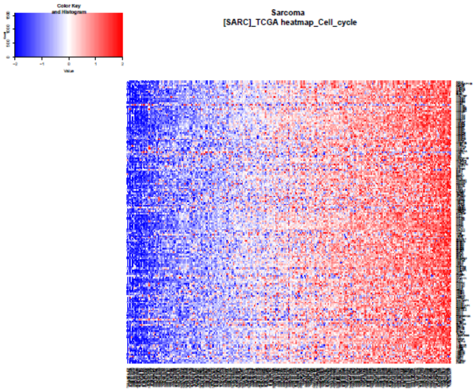

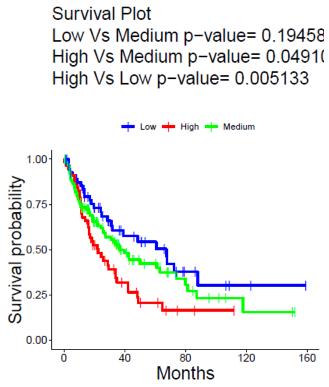

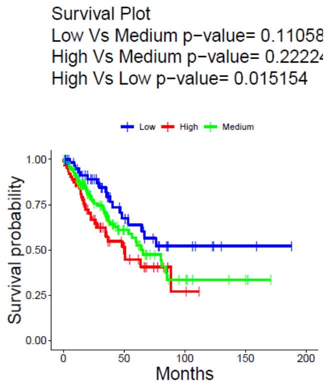

SKCM

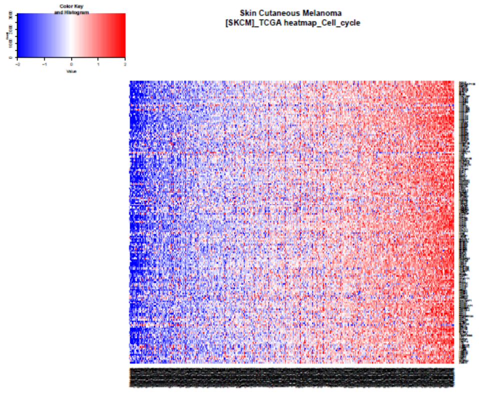

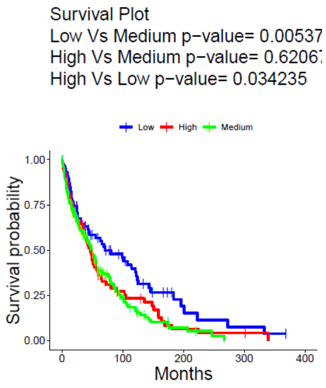

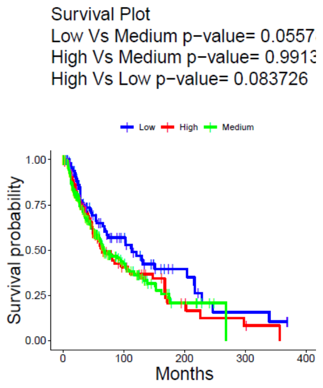

Disease Free Survial

Overall Survival

STAD

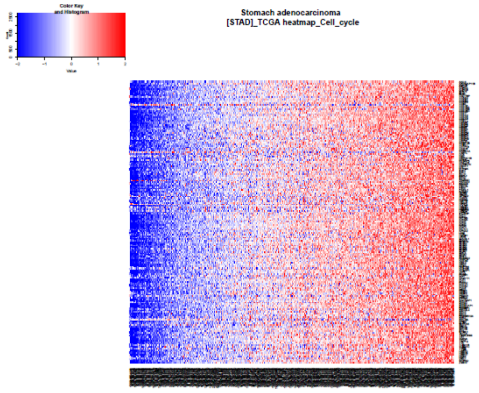

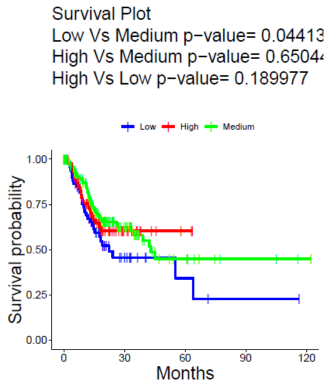

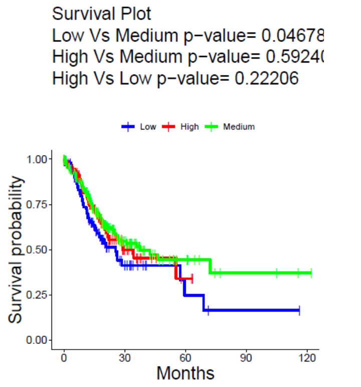

TGCT

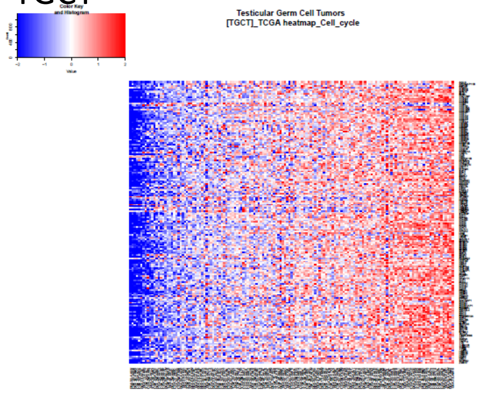

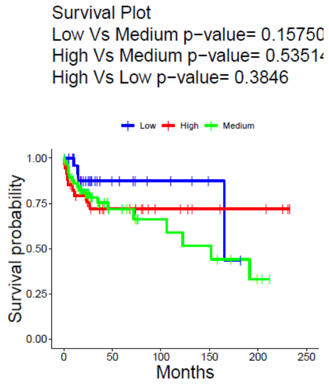

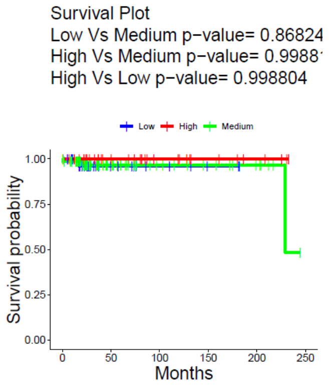

THYM

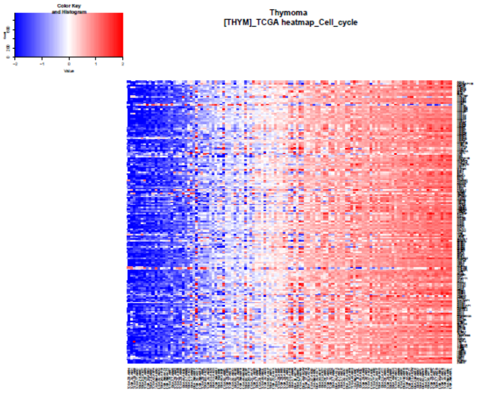

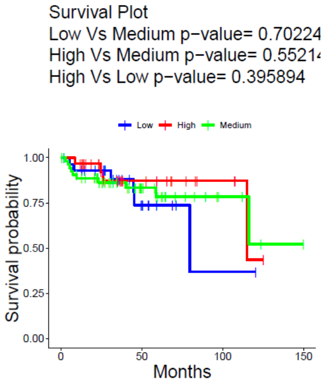

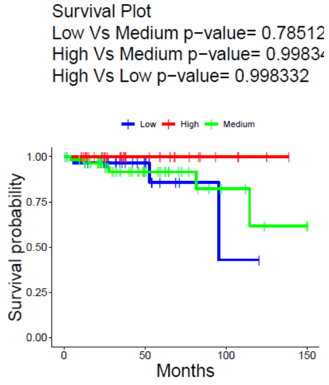

THCA

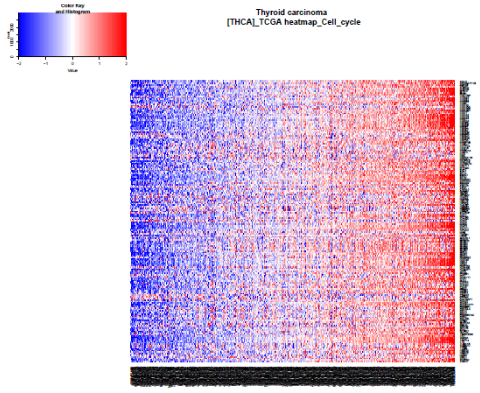

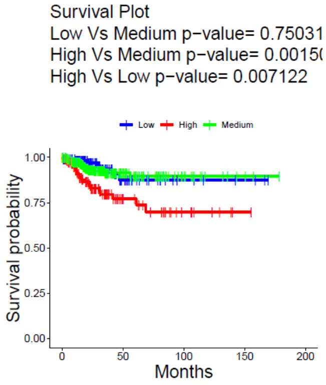

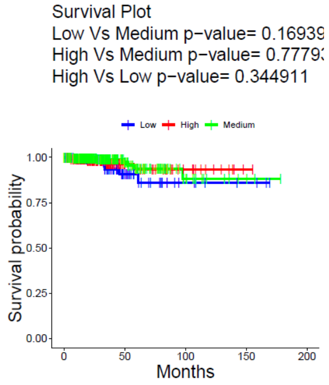

	Disease Free Survial	Overall Survival
UCS		

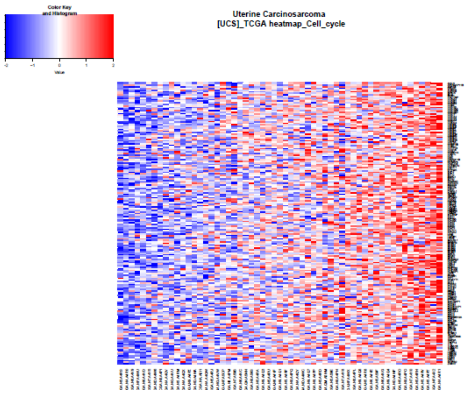

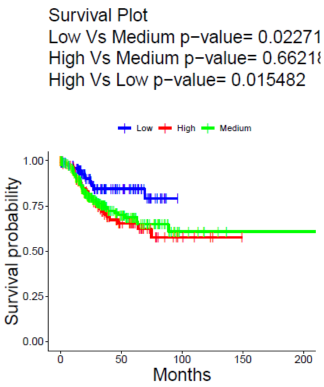

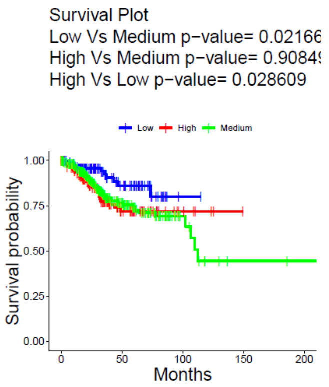

UCEC

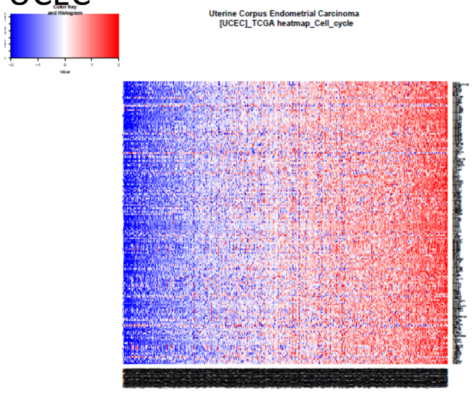

NA

NA

UVM

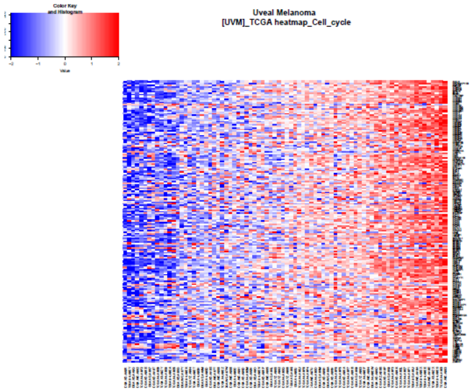

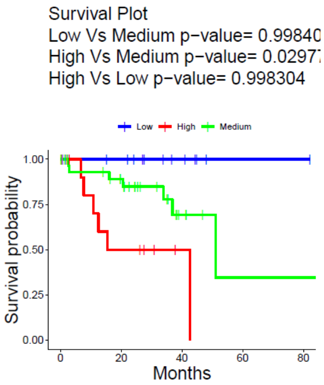

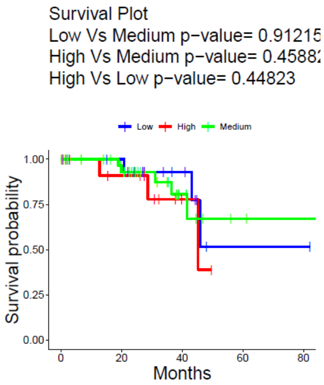

Supplementary Figure 14: Heatmaps illustrate the behavior of the CDK4/6-RB integrated signature across all tumors in the pan-cancer data set clustered based on average signature value. The corresponding Kaplan-Meier analysis is based on the average CDK4/6-RB integrated signature: high 25%, medium 50%, and low 25%. Statistical significance was determined by log-rank test. Both the disease-free survival and overall survival results are shown for all cases for which the data is available. Table summarizes the mean and median overall and disease-free survival of different tumors types in the TCGA pan-cancer cohort. NA indicates that survival data is not available for that tumor type.

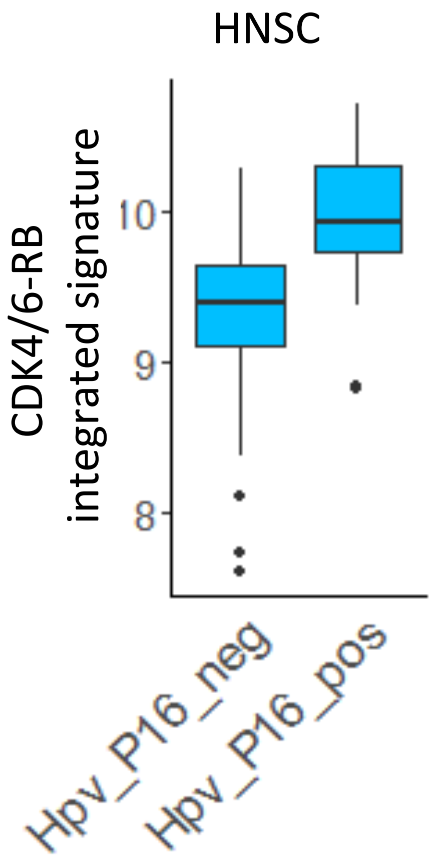

Supplementary Figure 15: Expression of the CDK4/6-RB integrated signature in HNSC HPV p16-positive (N=41) vs. HPV p16-negative cases (N=74). The statistical significance was determined by two-sided Student's t-test.

Supplementary Figure 16

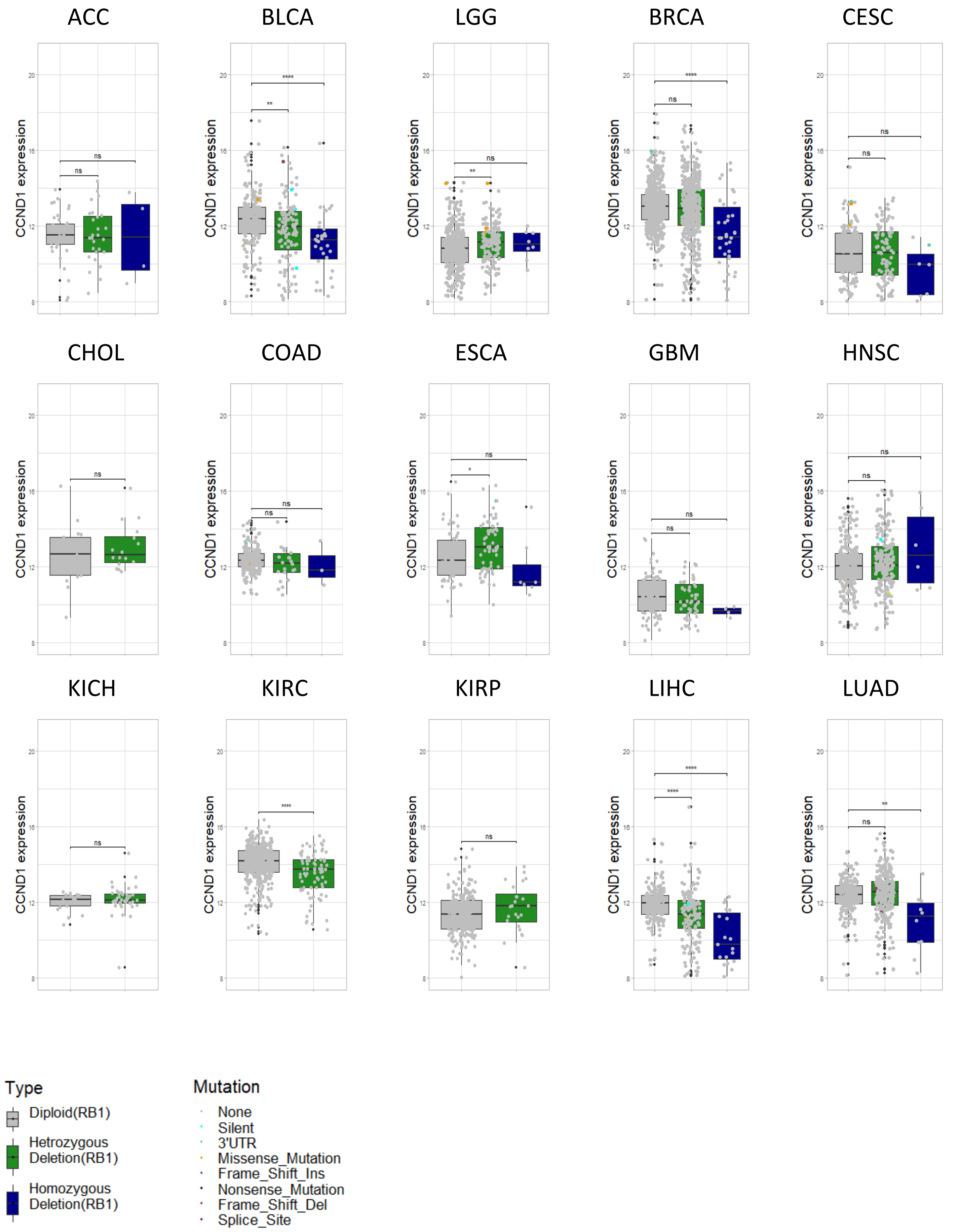

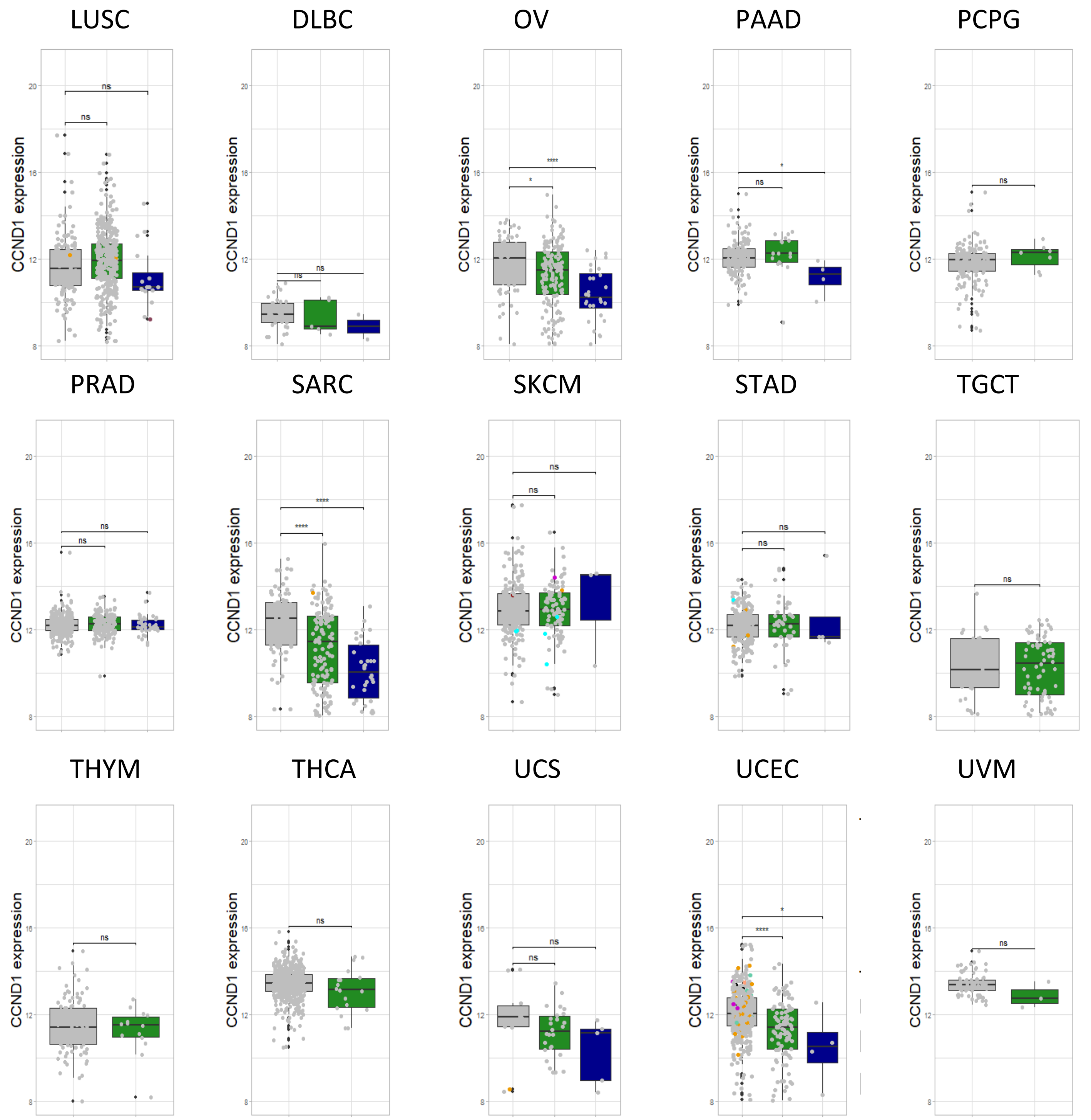

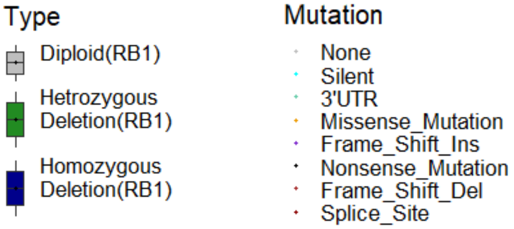

Supplementary Figure 16: Analysis of CCND1 expression in tumors exhibiting diploid, heterozygous loss, or deletion of the RB1 locus. The data from each tumor type and all cases is summarized in the bar plots and statistical significance was determined by two-sided Student’s t-test (*p<0.05**p<0.01, ***p<0.001, ****p<0.0001).

RB1 deletion vs. wild-type

Term	P-value	Adjusted P-value
DNA metabolic process (GO:0006259)	1.77E-36	9.04E-33
DNA replication (GO: 0006260)	1.28E-31	3.26E-28
G1/S transition of mitotic cell cycle (GO:0000082)	5.52E-30	9.39E-27
mitotic cell cycle phase transition (GO:0044772)	1.82E-25	2.33E-22
cell cycle G1/S phase transition (GO:0044843)	2.84E-23	2.90E-20
mitotic sister chromatid segregation (GO:0000070)	1.52E-19	1.29E-16
cellular macromolecule biosynthetic process (GO: 0034645)	2.02E-19	1.47E-16
mitotic spindle organization (GO:0007052)	9.13E-19	5.83E-16
DNA repair (GO:0006281)	1.27E-17	7.20E-15
DNA-dependent DNA replication (GO:0006261)	1.68E-17	8.59E-15
**Term**	**P-value**	**Adjusted P-value**
E2F4_ENCODE	1.35E-146	1.41E-144
FOXM1_ENCODE	2.19E-38	1.14E-36
NFYB_ENCODE	1.67E-31	5.80E-30
E2F6_ENCODE	1.81E-30	4.70E-29

RB1 single copy loss vs. wild-type

Term	P-value	Adjusted P-value
mitotic sister chromatid segregation (GO:0000070)	2.37E-23	1.21E-19
mitotic spindle organization (GO:0007052)	8.93E-19	2.28E-15
metaphase plate congression (GO:0051310)	2.18E-16	3.70E-13
regulation of mitotic cell cycle phase transition (GO: 1901990)	8.62E-16	1.10E-12
mitotic metaphase plate congression (GO:0007080)	1.20E-14	1.22E-11
mitotic nuclear division (GO: 0140014)	9.31E-14	7.92E-11
**Term**	**P-value**	**Adjusted P-value**
E2F4_ENCODE	9.62E-95	1.00E-92
FOXM1_ENCODE	3.06E-43	1.59E-41
SIN3A_ENCODE	5.49E-30	1.90E-28
NFYA_ENCODE	4.88E-23	1.27E-21

Supplementary Figure 17: Gene ontology and enriched transcription factor binding sites of genes consistently up-regulated with homozygous deletion or heterozygous loss of RB1. The analysis was performed using ENRICHR and top terms are summarized.

Supplementary Figure 18

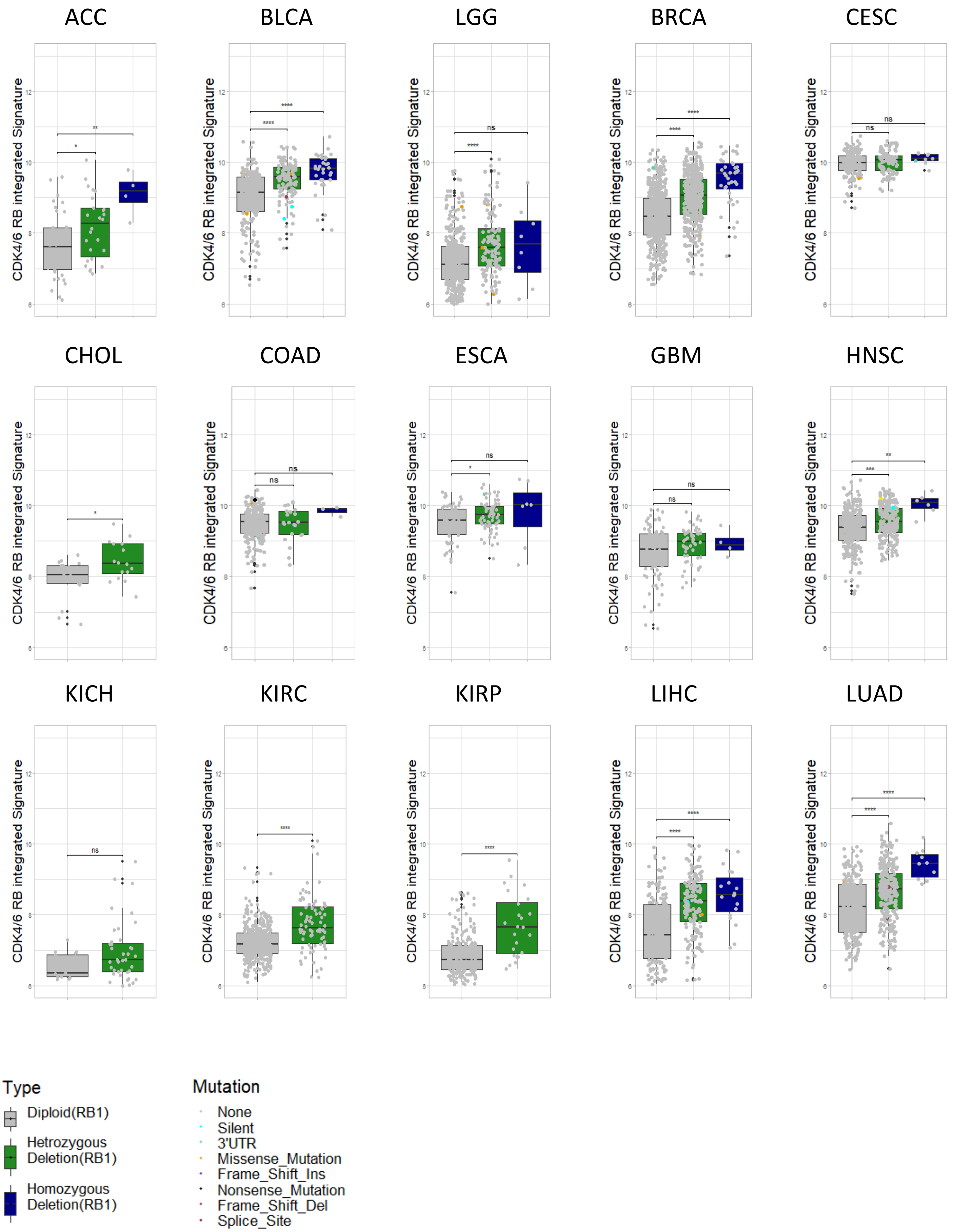

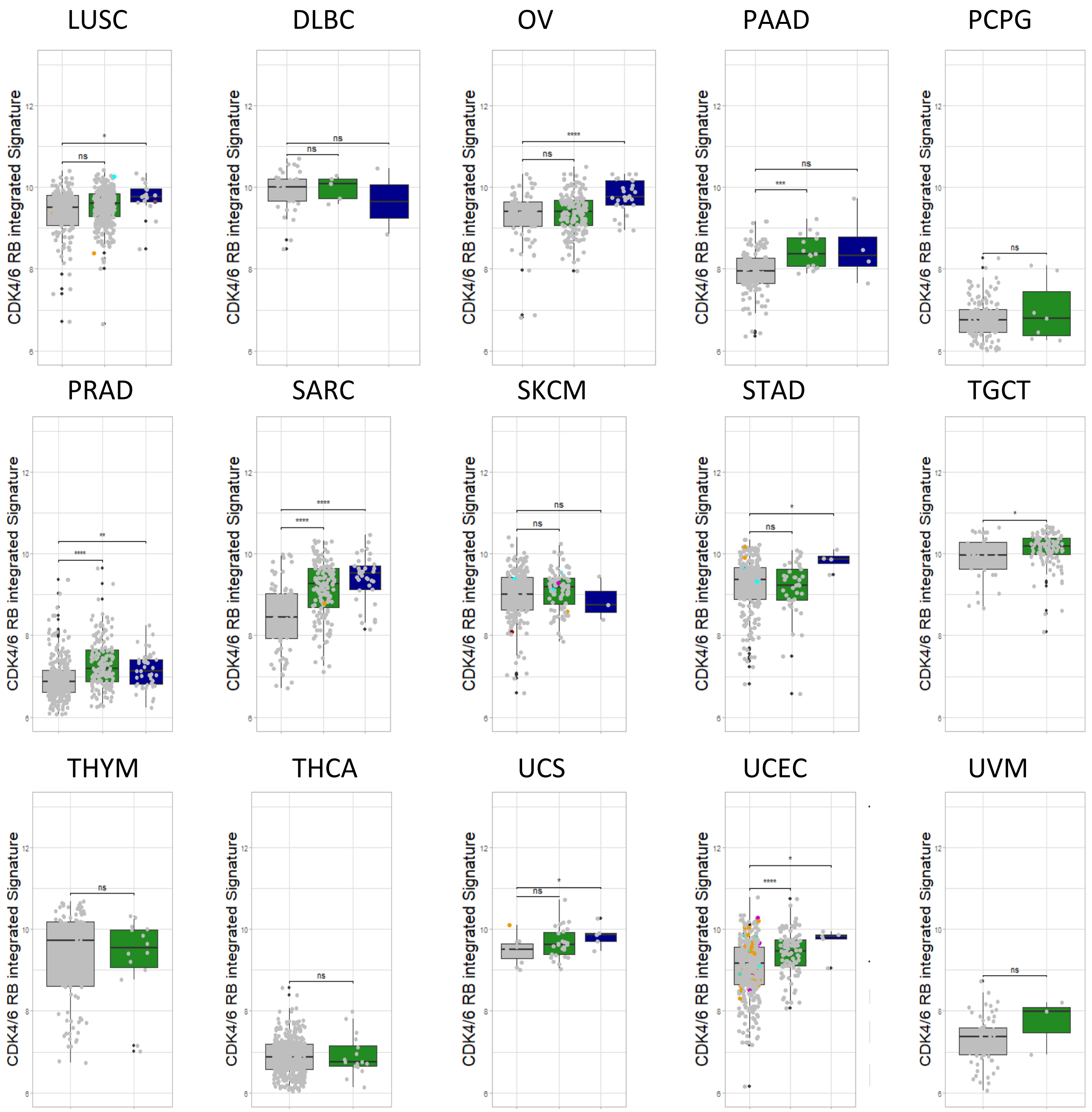

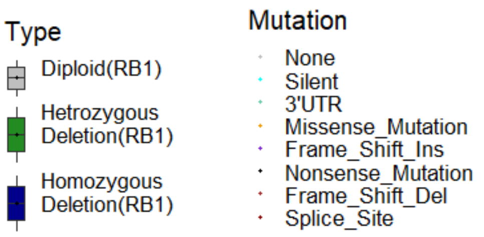

Supplementary Figure 18: Analysis of CDK4/6 RB integrated signature expression in tumors exhibiting diploid, heterozygous loss, or deletion of the RB1 locus. The data from each tumor type and all cases is summarized in the bar plots and statistical significance was determined by two-sided Student's t-test (*p<0.05**p<0.01, ***p<0.001). Legend denotes different forms of RB1 gene alterations beyond copy number changes

Supplementary Figure 19

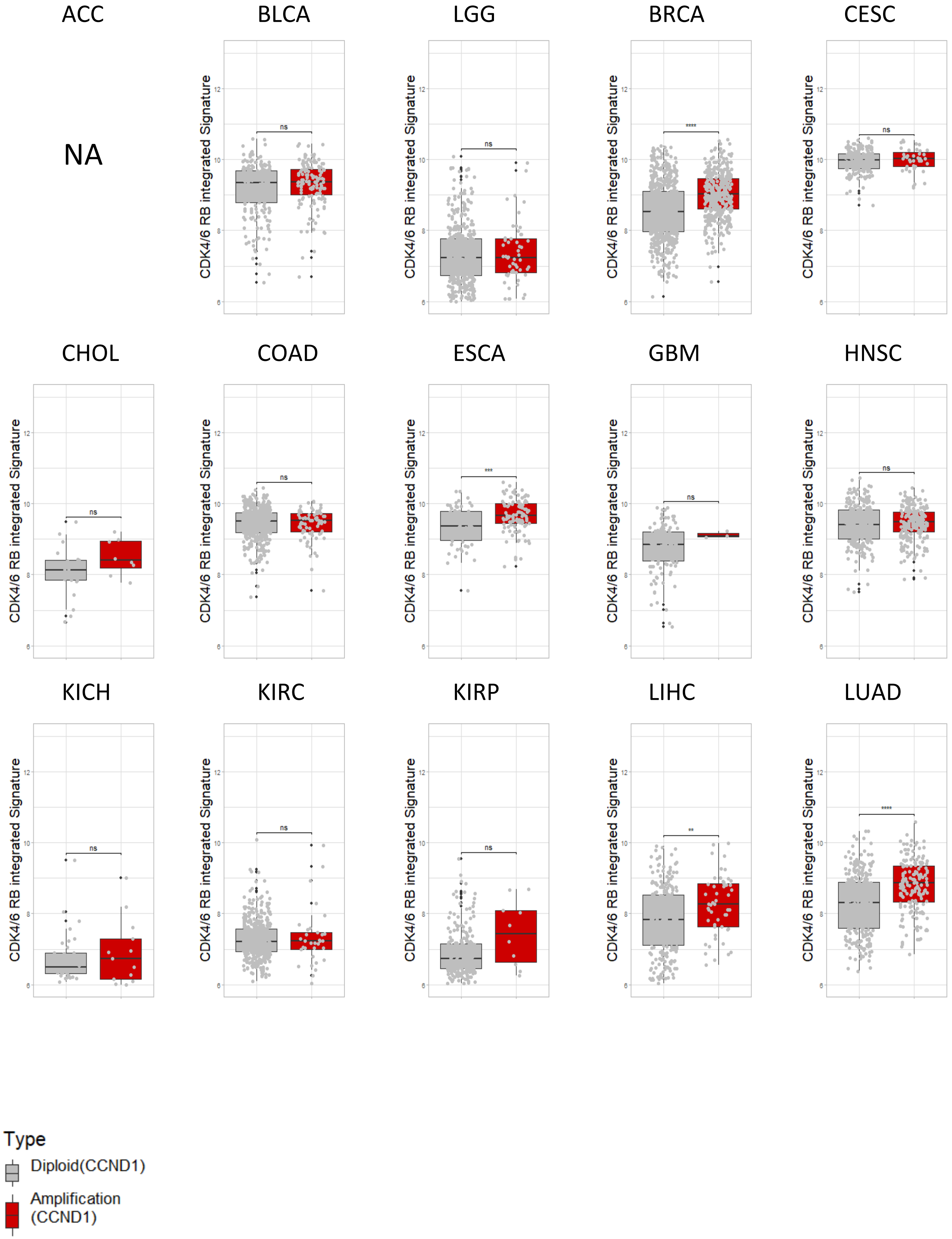

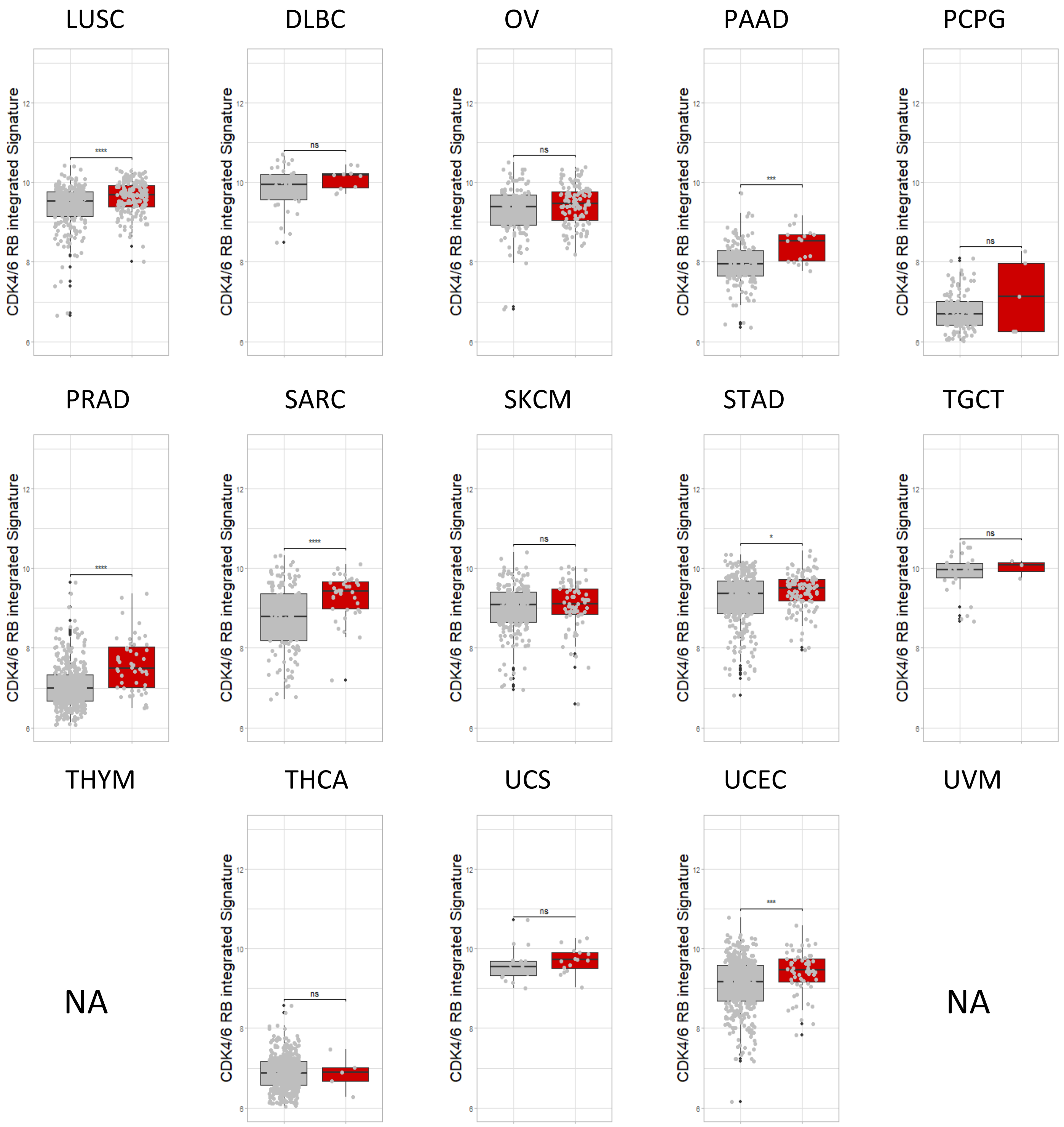

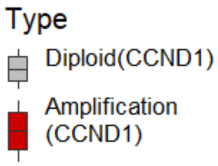

Supplementary Figure 19: Analysis of the CDK4/6 RB integrated signature stratified by amplification of CCND1. The number of cases in each group is shown. Statistical significance was determined by two-sided Student's t-test.

Supplementary Figure 20

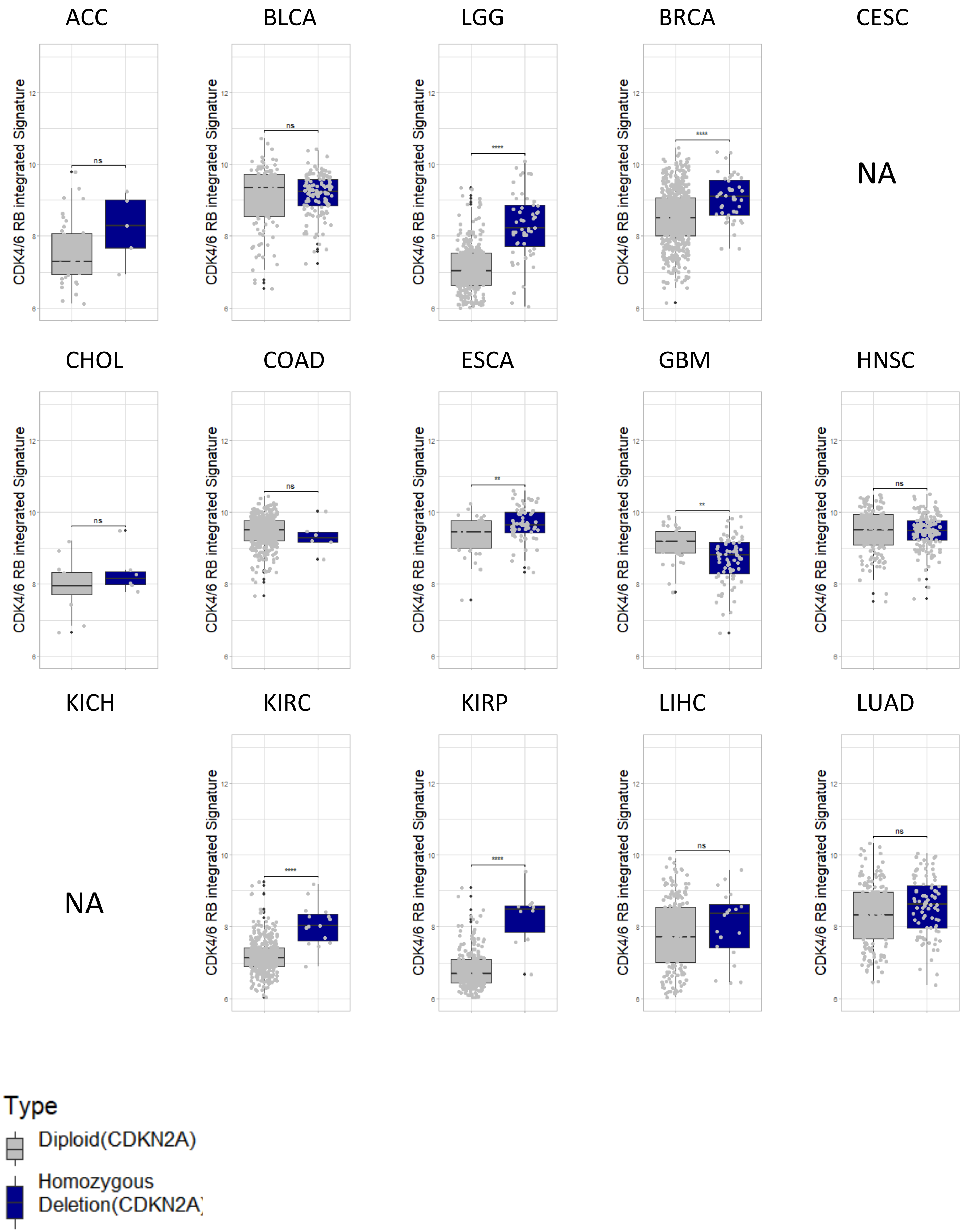

Supplementary Figure 20

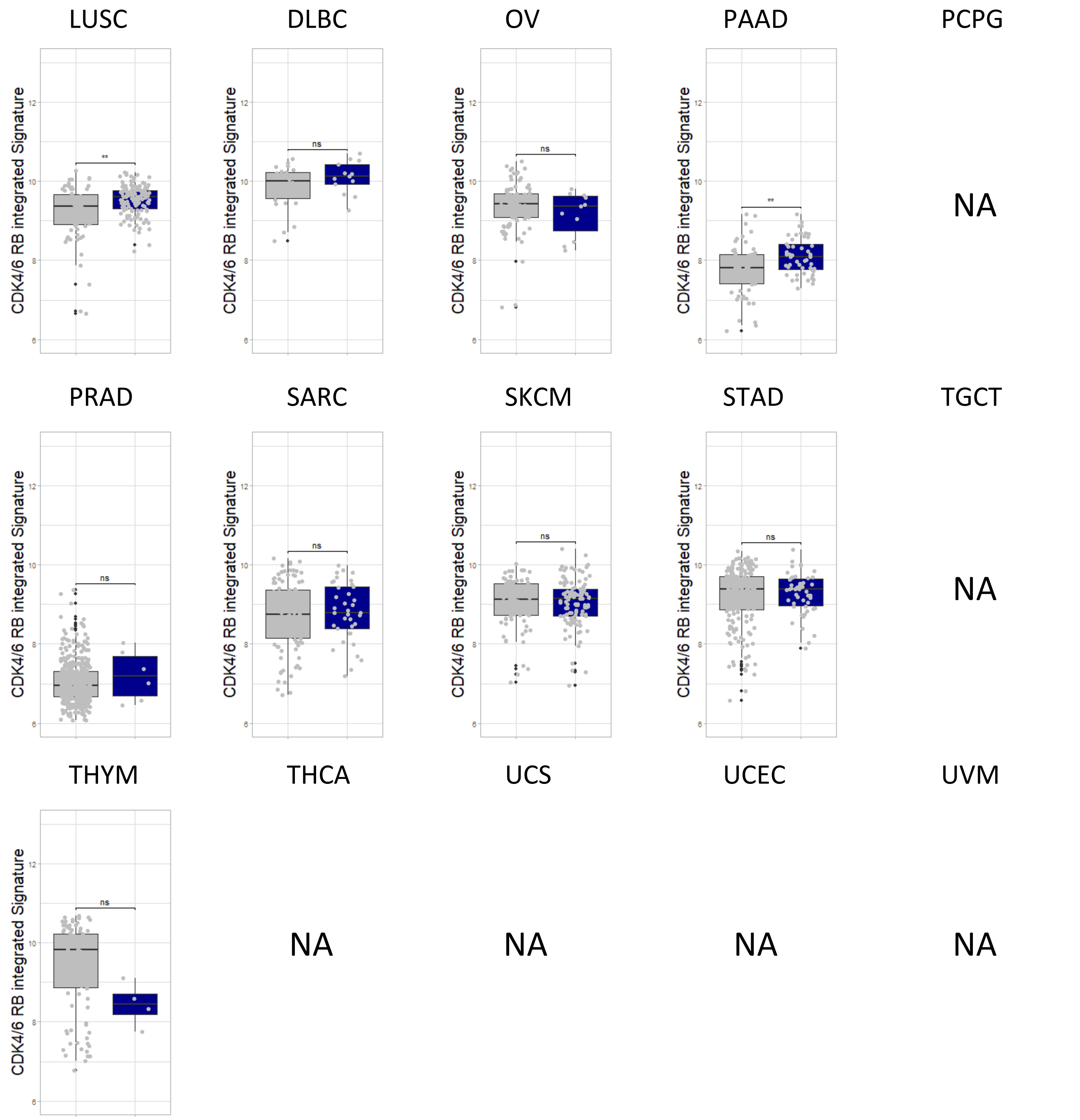

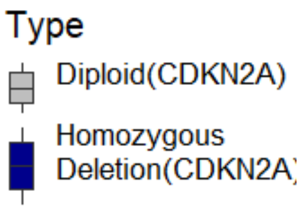

Supplementary Figure 20: Analysis of the CDK4/6 RB integrated signature stratified by deletion of CKDN2A. The number of cases in each group is shown. Statistical significance was determined by two-sided Student's t-test.

Supplementary Figure 21

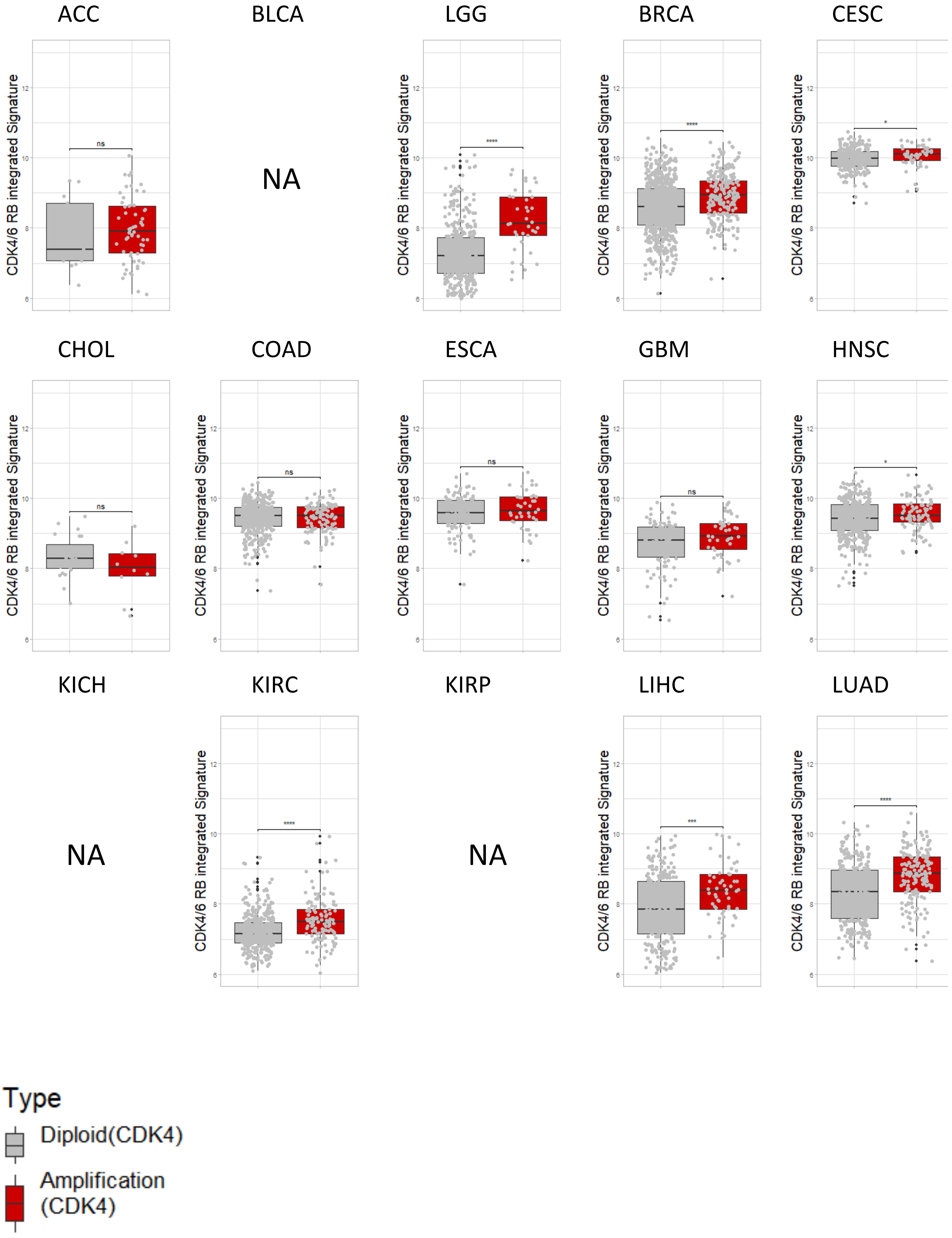

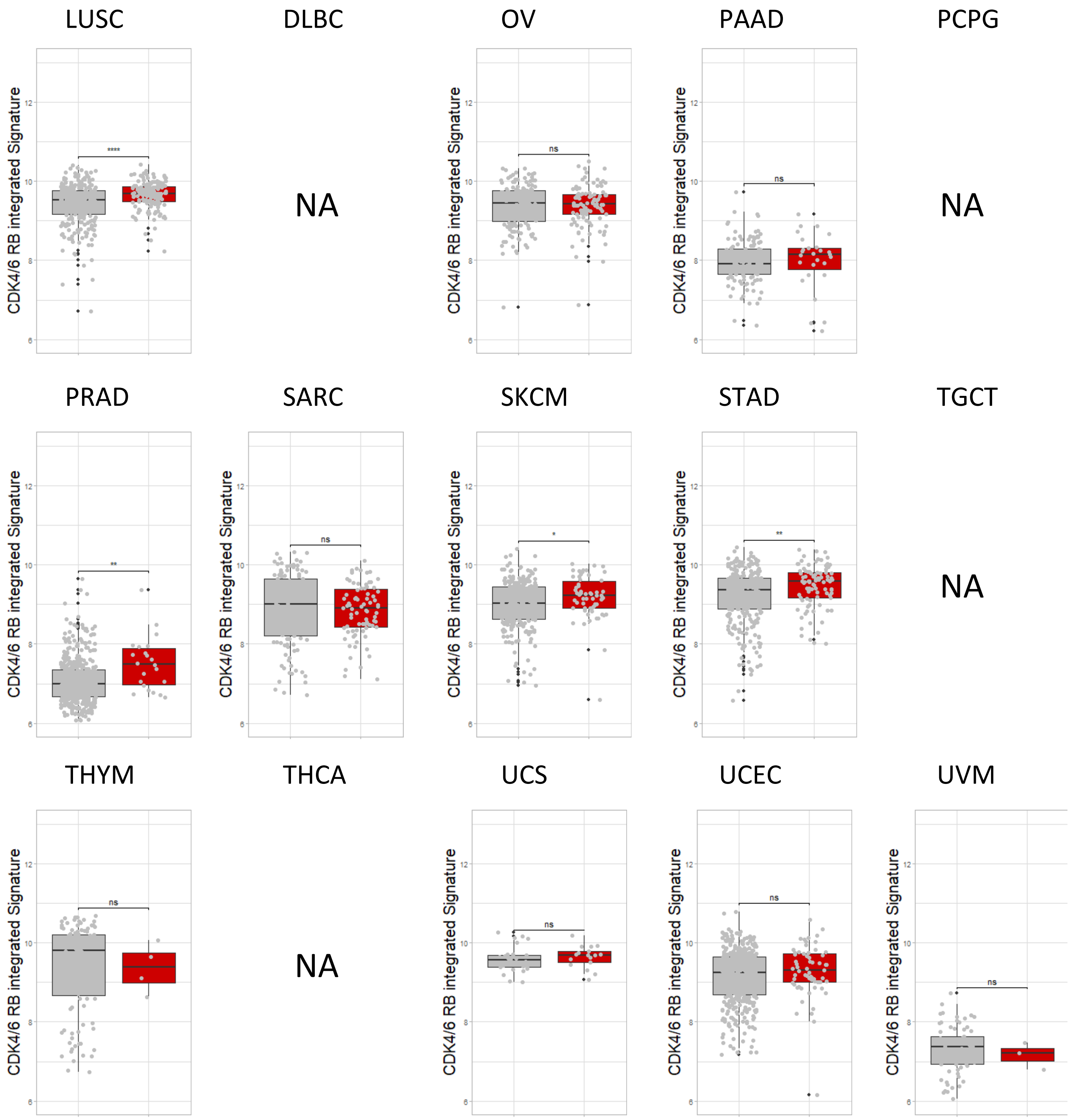

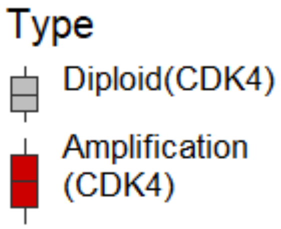

Supplementary Figure 21: Analysis of the CDK4/6 RB integrated signature stratified by amplification of CDK4. The number of cases in each group is shown. Statistical significance was determined by two-sided Student's t-test.

Supplementary Figure 22

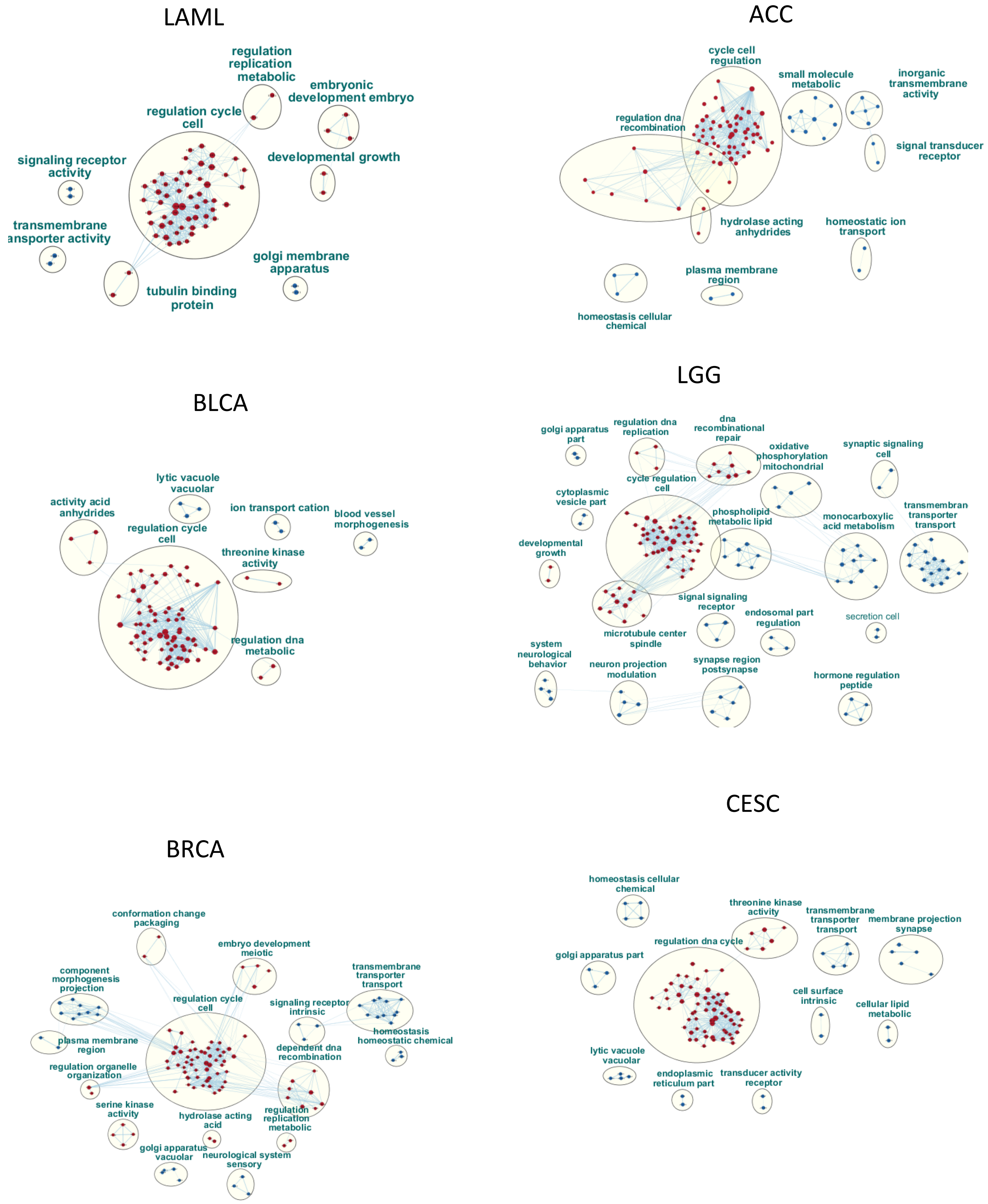

Supplementary Figure 22

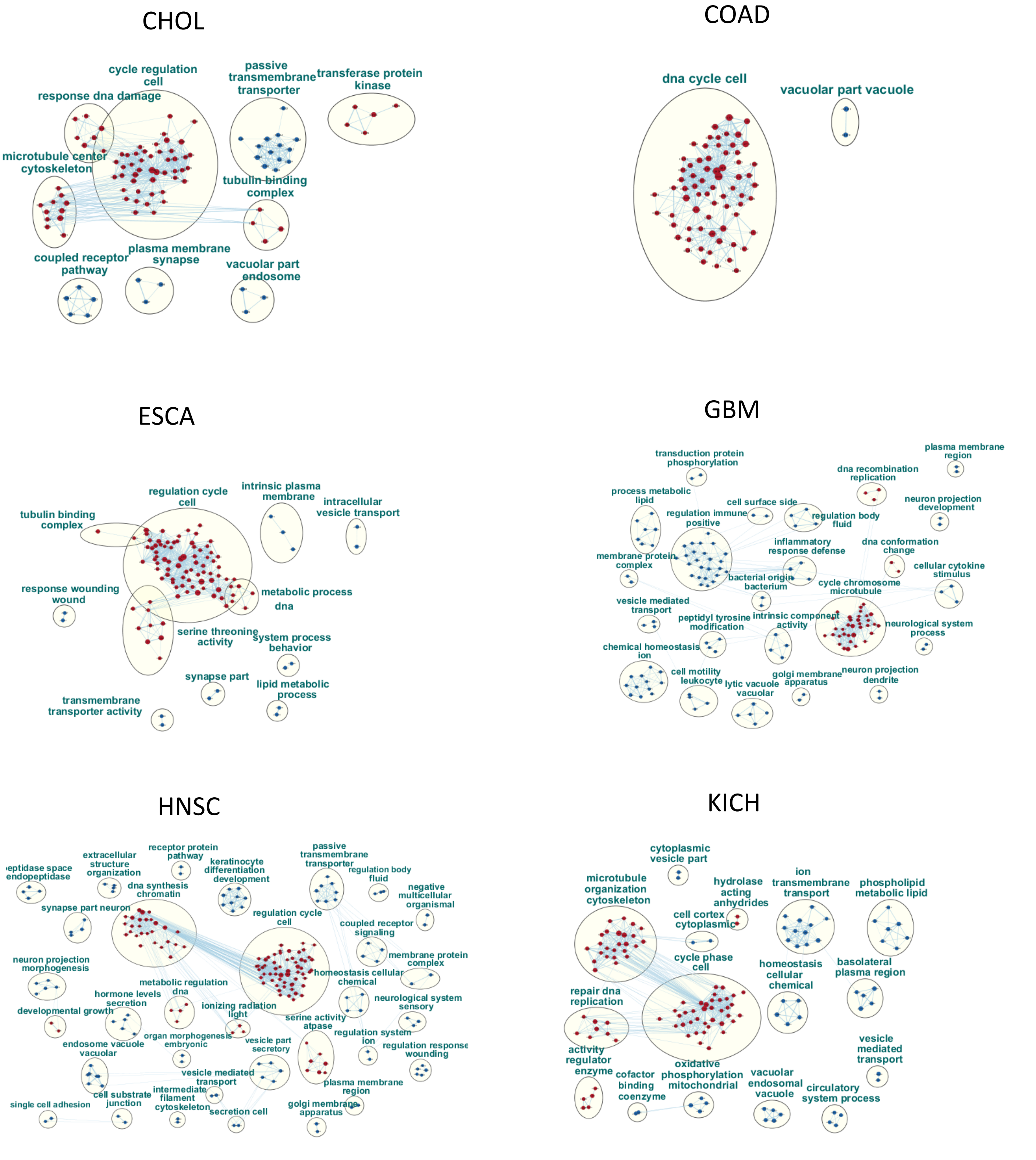

Supplementary Figure 22

KIRC

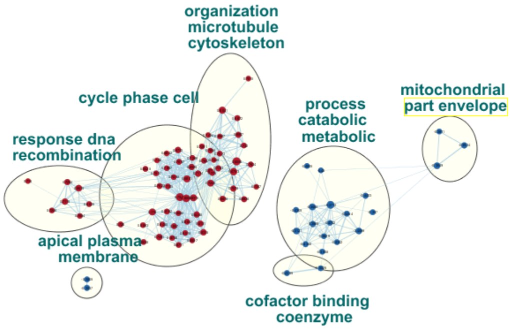

KIRP

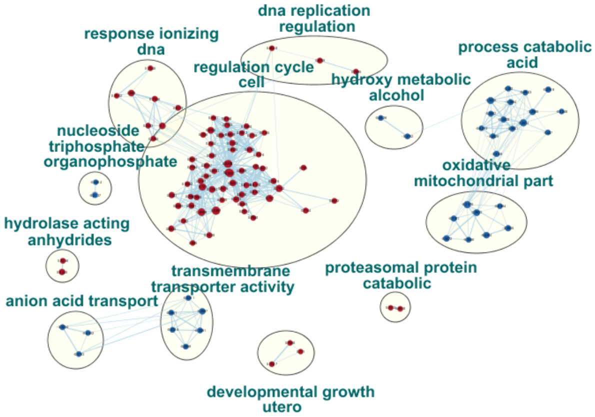

LIHC

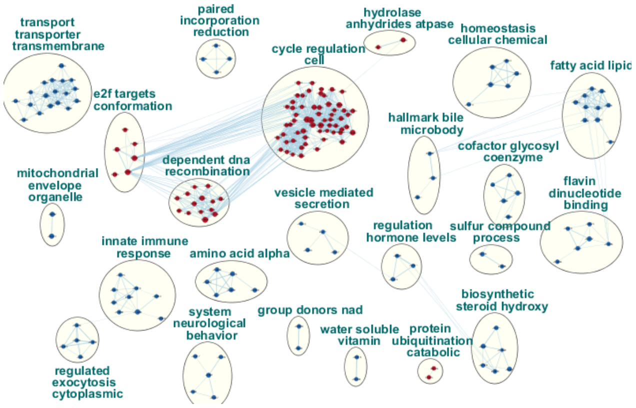

LUAD

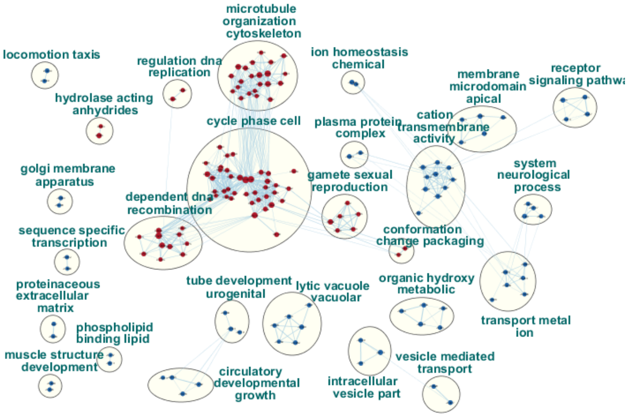

LUSC

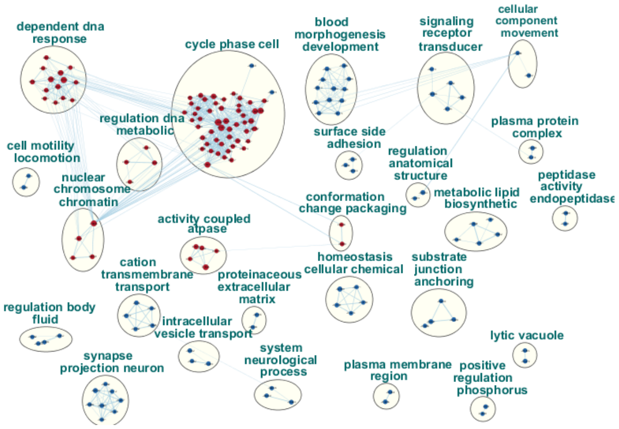

DLBC

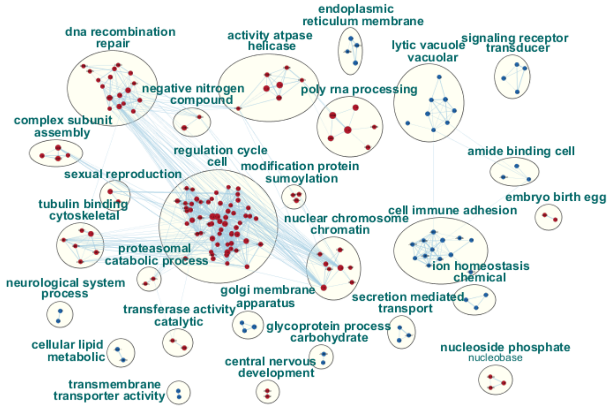

Supplementary Figure 22

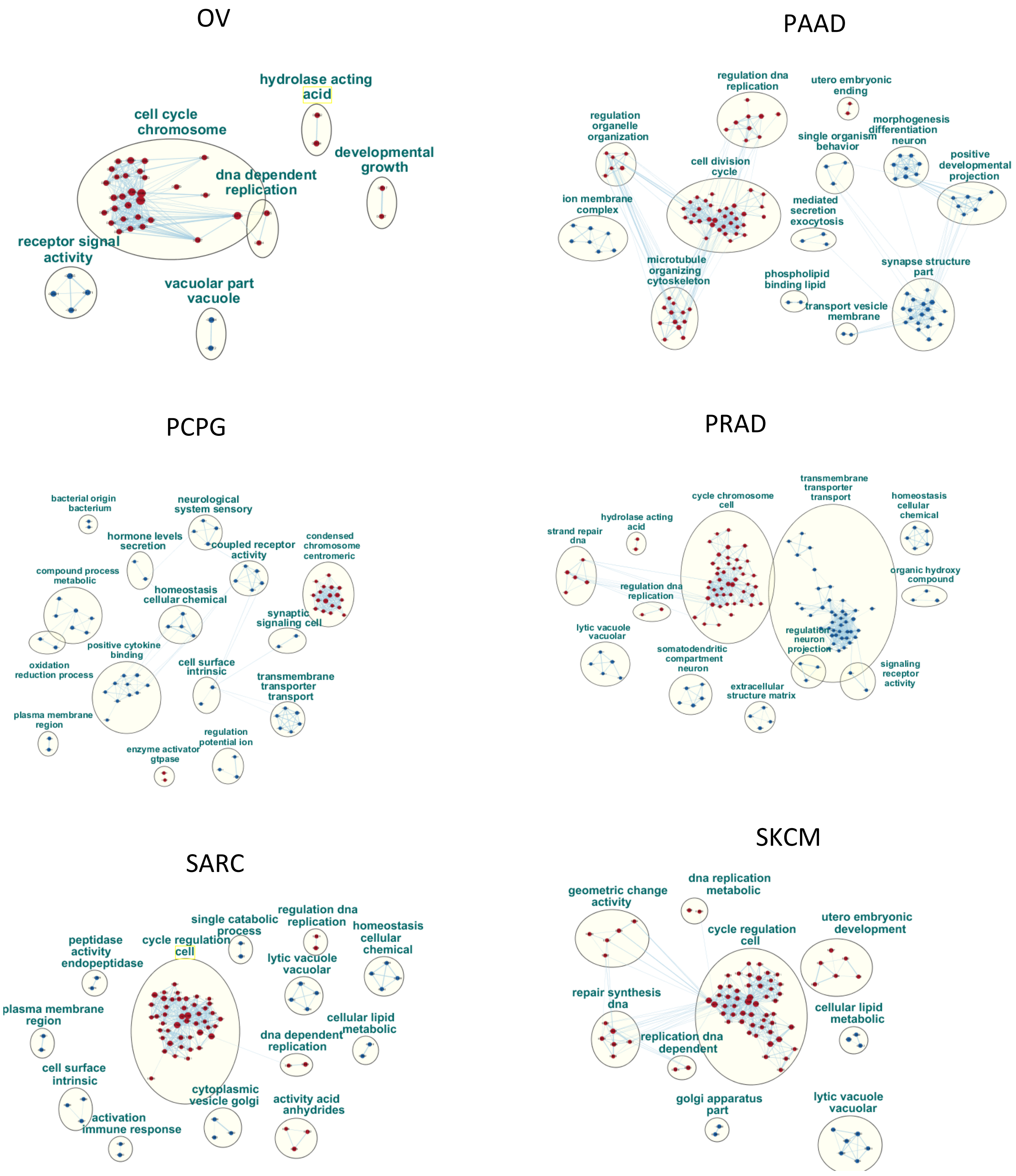

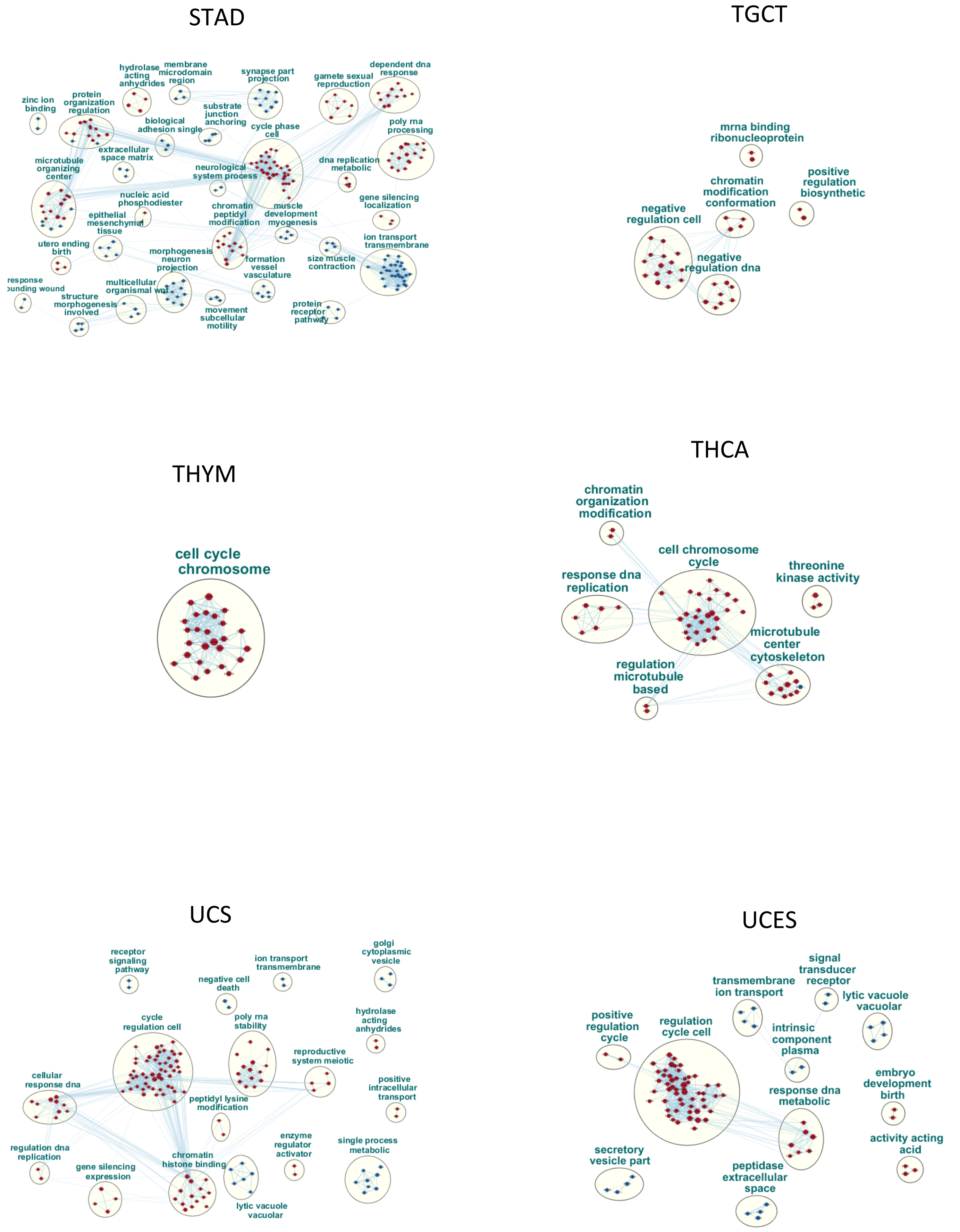
STAD
hydrolase acting anhydrides
membrane microdomain region
synapse part projection
dependent dna response
gamete sexual reproduction
zinc ion binding
protein organization regulation
substrate junction anchoring
biological adhesion single
cycle phase cell
poly rna processing
extracellular space matrix
microtubule organizing center
neurological system process
dna replication metabolic
nucleic acid phosphodiester
gene silencing localization
epithelial mesenchymal tissue
chromatin peptidyl modification
muscle development myogenesis
ion transport transmembrane
utero ending birth
morphogenesis neuron projection
formation vessel vasculature
size muscle contraction
response
multicellular organismal wnt
structure morphogenesis involved
movement subcellular motility
protein receptor pathway
TGCT
mrna binding ribonucleoprotein
chromatin modification conformation
positive regulation biosynthetic
negative regulation cell
negative regulation dna
THYM
cell cycle chromosome
THCA
chromatin organization modification
cell chromosome cycle
response dna replication
threonine kinase activity
microtubule center cytoskeleton
regulation microtubule based
UCS
receptor signaling pathway
ion transport transmembrane
golgi cytoplasmic vesicle
negative cell death
cycle regulation cell
poly rna stability
hydrolase acting anhydrides
reproductive system meiotic
cellular response dna
peptidyl lysine modification
positive intracellular transport
regulation dna replication
enzyme regulator activator
single process metabolic
gene silencing expression
chromatin histone binding
lytic vacuole vacuolar
UCES
signal transducer receptor
transmembrane ion transport
lytic vacuole vacuolar
positive regulation cycle
regulation cycle cell
intrinsic component plasma
embryo development birth
response dna metabolic
activity acting acid
secretory vesicle part
peptidase extracellular space

UVM

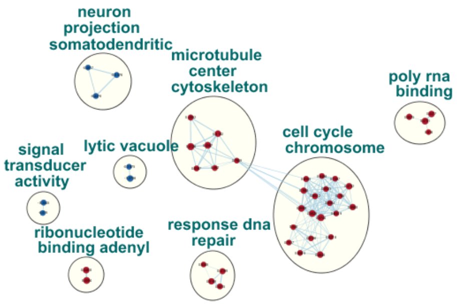

Supplementary Figure 22: Network maps of gene defined as positively (red) or inversely (blue) correlated with the CDK4/6-RB integrated signature in the individual tumor types. Genes were identified using bootstrapping to define significantly associated genes. Networks were developed using ranked gene set enrichment analysis and Cytoscape.

Term	P-value
MYC_ENCODE	1.22E-80
E2F4_ENCODE	1.81E-76
MAX_ENCODE	1.81E-62
TAF1_ENCODE	2.12E-59
E2F4_ENCODE	1.32E-76
SIN3A_ENCODE	3.34E-15
NFYB_ENCODE	2.22E-14
E2F6_ENCODE	2.38E-13
MAX_ENCODE	1.25E-54
MYC_ENCODE	5.28E-39
TAF1_ENCODE	1.79E-31
E2F6_ENCODE	1.70E-30

Term	P-value
RUNX1_CHEA	1.69E-04
IRF8_CHEA	4.13E-04
GATA1_CHEA	8.60E-04
GATA2_CHEA	0.0044
SUZ12_CHEA	2.41E-16
GATA2_CHEA	4.33E-09
ESR1_CHEA	6.83E-09
AR_CHEA	4.41E-08

Supplementary Figure 23: Enrichment of transcription factor binding sites of genes in the clusters of correlated genes from Figure 5B. Analysis was performed using ENRICHR and top terms and associated p-values are summarized in the table.

A	B	Neither	A Not B	B Not A	Both	Log2 Odds Ratio	p-Value	q-Value	Tendency
CDKN2A	CDKN2B	8475	435	23	1256	>3	<0.001	<0.001	Co-occurrence
CCND1	CDKN2A	8048	450	1447	244	1.593	<0.001	<0.001	Co-occurrence
CCND1	CDKN2B	8387	523	1108	171	1.307	<0.001	<0.001	Co-occurrence
RB1	RBL2	9247	706	174	62	2.222	<0.001	<0.001	Co-occurrence
RBL1	RBL2	9685	268	205	31	2.45	<0.001	<0.001	Co-occurrence
RB1	CDKN2A	7794	704	1627	64	-1.199	<0.001	<0.001	Mutual exclusivity
RB1	CDKN2B	8189	721	1232	47	-1.207	<0.001	<0.001	Mutual exclusivity
RB1	RBL1	9176	714	245	54	1.502	<0.001	<0.001	Co-occurrence
CCND2	CDK4	9671	234	257	27	2.118	<0.001	<0.001	Co-occurrence
CDKN2B	CDKN2C	8818	1237	92	42	1.702	<0.001	<0.001	Co-occurrence
CCND1	CDK6	9284	651	211	43	1.539	<0.001	<0.001	Co-occurrence
CCND2	RBL2	9715	238	213	23	2.14	<0.001	<0.001	Co-occurrence
CDK6	CDKN2A	8317	181	1618	73	1.052	<0.001	<0.001	Co-occurrence
RB1	CDKN2C	9313	742	108	26	1.595	<0.001	<0.001	Co-occurrence
CDK6	CDKN2B	8713	197	1222	57	1.045	<0.001	<0.001	Co-occurrence
CDKN2A	CDKN2C	8407	1648	91	43	1.269	<0.001	<0.001	Co-occurrence
CDK6	RBL2	9717	236	218	18	1.765	<0.001	<0.001	Co-occurrence
CCND2	RB1	9198	223	730	38	1.102	<0.001	<0.001	Co-occurrence
CCND1	RBL2	9291	662	204	32	1.139	<0.001	<0.001	Co-occurrence
CCND1	CDK4	9246	659	249	35	0.98	<0.001	0.001	Co-occurrence
CCND1	RBL1	9232	658	263	36	0.941	<0.001	0.002	Co-occurrence
CCND2	RBL1	9647	243	281	18	1.347	<0.001	0.002	Co-occurrence
CCND2	CDK6	9690	245	238	16	1.411	<0.001	0.002	Co-occurrence
CCND3	CDK4	9679	226	268	16	1.354	0.001	0.003	Co-occurrence
CDK6	RBL1	9653	237	282	17	1.296	0.001	0.003	Co-occurrence
CCND1	RB1	8760	661	735	33	-0.749	0.002	0.004	Mutual exclusivity
CDKN2B	CDKN2D	8742	1269	168	10	-1.286	0.002	0.004	Mutual exclusivity
CCND3	RBL1	9664	226	283	16	1.274	0.002	0.005	Co-occurrence
CDKN2A	CDKN2D	8336	1675	162	16	-1.025	0.002	0.005	Mutual exclusivity
CDK4	RBL2	9684	269	221	15	1.289	0.003	0.006	Co-occurrence
CCND2	CDKN2A	8298	200	1630	61	0.635	0.003	0.006	Co-occurrence
CCND2	CDKN2B	8697	213	1231	48	0.671	0.004	0.008	Co-occurrence
CDK4	RBL1	9623	267	282	17	1.119	0.004	0.008	Co-occurrence
CCND1	CCND2	9262	666	233	28	0.741	0.011	0.021	Co-occurrence
CDK4	CDKN2C	9780	275	125	9	1.356	0.013	0.024	Co-occurrence
RB1	CDKN2D	9265	746	156	22	0.809	0.015	0.027	Co-occurrence
CCND2	CDKN2C	9802	253	126	8	1.299	0.022	0.038	Co-occurrence
CCND2	CCND3	9698	249	230	12	1.023	0.022	0.038	Co-occurrence
CCND3	RBL2	9722	231	225	11	1.041	0.025	0.043	Co-occurrence
CDKN2C	CDKN2D	9883	128	172	6	1.429	0.03	0.049	Co-occurrence
RBL2	CDKN2C	9826	229	127	7	1.242	0.036	0.058	Co-occurrence
CCND3	RB1	9205	216	742	26	0.578	0.042	0.065	Co-occurrence
CDK6	RB1	9194	227	741	27	0.561	0.043	0.065	Co-occurrence
RBL1	CDKN2C	9764	291	126	8	1.091	0.044	0.065	Co-occurrence
CDK4	CDK6	9663	272	242	12	0.817	0.052	0.076	Co-occurrence
CDK4	CDKN2D	9736	275	169	9	0.915	0.061	0.087	Co-occurrence
CCND3	CDKN2D	9777	234	170	8	0.975	0.062	0.087	Co-occurrence
CCND1	CCND3	9276	671	219	23	0.538	0.065	0.09	Co-occurrence
CCND1	CDKN2C	9375	680	120	14	0.686	0.072	0.097	Co-occurrence
CCND3	CDKN2A	8305	193	1642	49	0.361	0.075	0.099	Co-occurrence
CCND3	CDK6	9703	232	244	10	0.777	0.081	0.105	Co-occurrence
CCND3	CDKN2C	9819	236	128	6	0.964	0.1	0.126	Co-occurrence
RBL2	CDKN2B	8697	213	1256	23	-0.419	0.109	0.136	Mutual exclusivity
CCND1	CDKN2D	9333	678	162	16	0.443	0.155	0.19	Co-occurrence
CCND2	CDKN2D	9757	254	171	7	0.653	0.173	0.207	Co-occurrence
CDK4	RB1	9154	267	751	17	-0.366	0.188	0.222	Mutual exclusivity
RBL2	CDKN2D	9781	230	172	6	0.569	0.231	0.268	Co-occurrence
RBL1	CDKN2D	9719	292	171	7	0.446	0.268	0.305	Co-occurrence
CDK6	CDKN2D	9763	248	172	6	0.458	0.284	0.318	Co-occurrence
RBL1	CDKN2A	8252	246	1638	53	0.118	0.32	0.352	Co-occurrence
CDK6	CDKN2C	9803	252	132	2	-0.763	0.346	0.375	Mutual exclusivity
CDK4	CDKN2B	8663	247	1242	37	0.063	0.43	0.458	Co-occurrence
CDK4	CDKN2A	8260	238	1645	46	-0.043	0.466	0.484	Mutual exclusivity
RBL2	CDKN2A	8302	196	1651	40	0.037	0.469	0.484	Co-occurrence
RBL1	CDKN2B	8648	262	1242	37	-0.024	0.506	0.514	Mutual exclusivity
CCND3	CDKN2B	8698	212	1249	30	-0.021	0.519	0.519	Mutual exclusivity

Supplementary Table 1: Mutual exclusivity analysis of the indicated RB-pathway associated genes in the full TCGA pan-cancer cohort.
