# Supplementary material for: Pan-cancer molecular analysis of the RB tumor suppressor pathway
Source: Commun Biol. 2020 Apr 2;3:158. doi: 10.1038/s42003-020-0873-9 (PMC7118159; doi:10.1038/s42003-020-0873-9)
Supplement: Supplementary file 2 — Description of Additional Supplementary Files [file 42003_2020_873_MOESM2_ESM.pdf]

Description of additional supplementary files:

Supplementary Data 1: Spreadsheet listing the genes that encompass the CDK4/6-RB integrated signature.

Supplementary Data 2: Spreadsheet denotes genes that are differentially expressed based on the deletion of RB1. The tumor cases which exhibit >5% deletion of RB1 were used for this analysis. All significantly associated genes that are either up or down regulated with RB1 deletion are indicated by a "1" in the tumor types as listed in the separate tabs.

Supplementary Data 3: Spreadsheet denotes genes that are differentially expressed based on the heterozygous loss RB1. The tumor cases which exhibit >10% heterozygous loss of RB1 were used for this analysis. All significantly associated genes that are either up or down regulated with RB1 deletion are indicated by a "1" in the tumor types as listed in the separate tabs.

Supplementary Data 4: Spreadsheet lists all genes were identified as being either positively or inversely correlated with the CDK4/6-RB integrated signature. Cutoffs were determined by bootstrapping as defined in the Methods.
